# Directional guidance to orient Schwann cell alignment in nerve regeneration requires Plexin-B1

**DOI:** 10.1126/sciadv.adw4136

**Published:** 2026-04-24

**Authors:** Jiaxi Li, Haofei Ni, Dalia Halawani, Molly Estill, Aarthi Ramakrishnan, Chrystian Junqueira Alves, Li Shen, Xijing He, Roland H. Friedel, Hongyan Zou

**Affiliations:** ^1^ Department of Orthopedics, The Second Affiliated Hospital of Xi’an Jiaotong University, Xi’an, China.; ^2^ Nash Family Department of Neuroscience, Friedman Brain Institute, Icahn School of Medicine at Mount Sinai, New York, NY, USA.; ^3^ Department of Orthopedics, Tongji Hospital, Tongji University School of Medicine, Shanghai, China.; ^4^ Department of Orthopedics, Xi’an International Medical Center Hospital, Xi’an, China.; ^5^ Department of Neurosurgery, Icahn School of Medicine at Mount Sinai, New York, NY, USA.

## Abstract

Directional cues are essential for orienting cells during tissue morphogenesis and repair. In peripheral nerve regeneration, Schwann cells (SCs) align longitudinally in the nerve bridge to guide axonal pathfinding, but the mechanisms are not fully understood. We show here that after nerve injury, activated SCs up-regulate the guidance receptor Plexin-B1, enabling membrane plasticity required for SC polarization and longitudinal alignment along the axons. Aligned axon-SC provides positional cues to orient macrophages and extracellular matrix. Loss of Plexin-B1 disrupts SC morphological transformation, contact inhibition of locomotion between SCs, and axon-SC alignment, leading to SC misorientation, excessive inflammation, and delayed axon regeneration and functional recovery. These findings identify Plexin-B1 as a key orchestrator to orient SCs by regulating both SC-SC and axon-SC interactions during nerve repair. Elucidating the mechanisms of spatial guidance in nerve repair after injury has potential implications for therapeutic strategies to enhance neural regeneration.

## Teaser

Plexin-B1–mediated membrane remodeling is needed to orient Schwann cells in nerve repair.

## INTRODUCTION

Peripheral nerve injury represents a major clinical challenge. Current approaches to promoting nerve repair, including microsurgical reapproximation, autologous nerve grafting, implantation of bioengineered scaffolds, and cell transplantation all face limitations (*1*–*3*). Thus, a deeper understanding of the cellular and molecular mechanisms of nerve repair is needed to develop more effective therapeutic interventions.

Following nerve transection injury, a gap is formed between the two retracting nerve stumps. This gap is quickly filled through glial cell infiltration and deposition of extracellular matrix (ECM), generating connecting tissue termed nerve bridge (*1*, *4*, *5*). The nerve bridge is first populated by fibroblasts and immune cells (*6*, *7*), with important roles in orchestrating neovascularization (*8*). In the proximal and distal nerve stumps, Schwann cells (SCs), which normally ensheath axons, undergo dedifferentiation upon loss of axonal contact and acquire an activated repair phenotype (*9*, *10*). Activated SCs migrate into the nerve bridge in characteristic chain-like patterns, functioning as scaffolds to guide regenerating axons across the bridge (*11*–*13*). In the distal nerve stump, severed axons undergo Wallerian degeneration, while SCs form bands of Büngner serving as substrate for the regenerating axons (*14*–*16*).

Recent investigations have unveiled molecular mechanisms governing the directed migration of SCs upon nerve injury. These include the identification of CCL3 as a macrophage-derived chemotactic factor that guides SCs into the nerve bridge (*17*). In addition, repulsive axon guidance molecules of the Slit/Robo families have been shown to orchestrate SC collective migration via contact inhibition of locomotion (CIL) (*18*). Our own work on semaphorin/plexin axon guidance molecules has revealed that after nerve injury, macrophages robustly up-regulate Plexin-B2, which mediates CIL with axons to direct macrophage alignment within the nerve bridge (*19*).

Plexins and semaphorins are receptor/ligand pairs that control not only axonal wiring in neurodevelopment but also cell migration and other cellular interactions in both development and adult physiology (*20*–*24*). Mammalian genomes contain three Plexin-Bs (B1 to B3), which are activated by class 4 semaphorins, a family of six members expressed in diverse cell types (*25*). Our recent study demonstrated that under pathological conditions, Plexin-Bs are reengaged by glial cells to mediate cell movement and spatial organization in response to traumatic injury (*19*, *26*, *27*), neurodegeneration (*28*), and glioblastoma invasion (*29*). Since the directed migration of SCs out of transected nerve stumps resembles collective cell migration in neurodevelopment, we wondered whether Plexins might be involved in directing SCs to build regenerative tracks after nerve injury. Of note, Sema4F expression in SCs has been reported as a key mediator of SC-axon contacts to suppress SC proliferation in the context of neurofibromatosis (*30*).

Here, we reveal an up-regulation of Plexin-B1 in SCs following sciatic nerve injury. Through integration of culture assays, transcriptomics, live-cell imaging, and in vivo nerve regeneration studies, we show that Plexin-B1–mediated CIL between SCs and alignment with axons is essential mechanisms to orient SCs during nerve regeneration. The aligned SC cords in the nerve bridge, in turn, provide positional guidance for macrophages, ECM, and neovasculature, ensuring axon pathfinding along the original nerve axis. Notably, the cellular disarray in the nerve bridge caused by Plexin-B1 deletion is also associated with increased inflammation, further impeding tissue repair and functional recovery.

## RESULTS

### Plexin-B1 is up-regulated in SCs in response to nerve injury

**Fig. 1.**
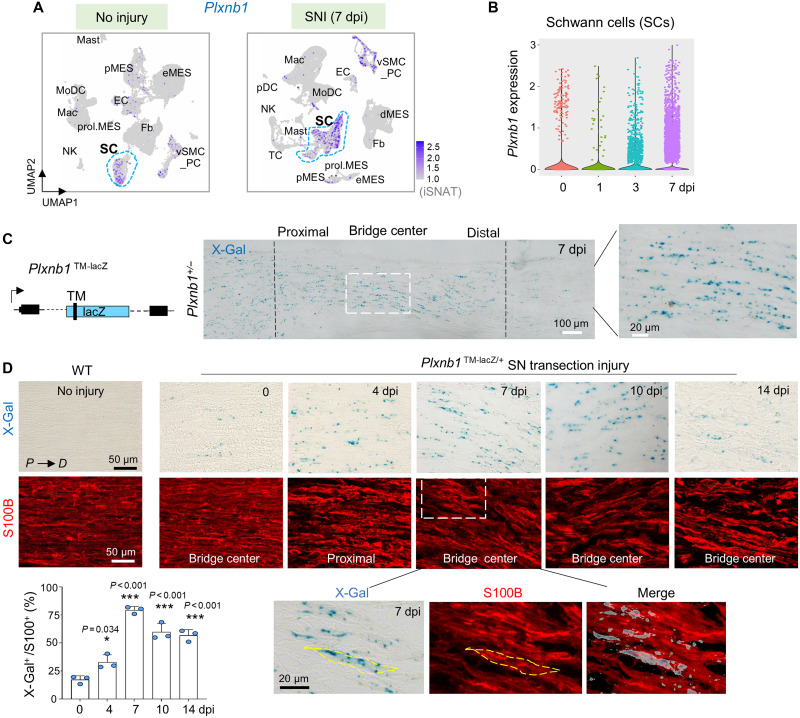
Plexin-B1 is up-regulated in SCs after sciatic nerve injury. (**A**) Uniform Manifold Approximation and Projection (UMAP) embedding of single-cell transcriptomes from noninjured and injured at 7 days posttransection injury (7 dpi) sciatic nerves, based on iSNAT platform data (*31*), showing induction of *Plxnb1* expression in SCs after injury. Expression is also detectable in vSMCs/PCs (vSMC_PC). NK, natural killer. (**B**) Violin plot illustrates *Plxnb1* up-regulation in SCs from 1 to 7 dpi after sciatic nerve injury. (**C**) Left: Schematic of *Plxnb1* transmembrane domain-lacZ reporter cassette (TM-lacZ) knockin. Right: X-galactosidase (X-Gal) staining of sagittal sciatic nerve section at 7 dpi shows expression of *Plxnb1*-lacZ at lesion center. (**D**) X-Gal staining combined with S100B immunostaining on sagittal sciatic nerve sections at 0 to 14 dpi. Note that the TM-lacZ reporter produces a punctate X-Gal staining pattern due aggregate formation. Bottom left: Quantification of fraction of X-Gal^+^ cells among S100B^+^ SCs. Data are means ± SEM; *n* = 3 mice per time point. One-way ANOVA with Dunnett’s multiple comparisons test. *P < 0.05 and ***P < 0.001. Mast, mast cells; eMES, endoneurial mesenchymal cells; pMES, perineurial mesenchymal cells; MoDC, monocyte-derived dendritic cells; EC, endothelial cells; Mac, macrophages; prol.MES, proliferating mesenchymal cells; Fb, fibroblasts; pDC, plasmacytoid dendritic cells; dMES, differentiating mesenchymal cells; TC, T cells.

To investigate the role of Plexin-B1 in peripheral nerve repair, we first assessed its expression dynamics using the injured sciatic nerve atlas (iSNAT), a single-cell RNA sequencing (RNA-seq) database of sciatic nerve at baseline and 1, 4, and 7 days postinjury (dpi) (*31*). In the uninjured nerve, *Plxnb1* was primarily expressed in SC; following sciatic nerve crush injury, *Plxnb1* expression was markedly up-regulated in SCs, as well as in pericytes (PCs) and vascular smooth muscle cells (vSMCs) (Fig. 1, A and B, and fig. S1A). Time course analysis showed a robust *Plxnb1* up-regulation in SCs at 3 and 7 dpi (Fig. 1B). We also surveyed the expression of *Sema4* genes, the canonical ligands for Plexin-B1. This revealed wide expression in diverse cell types in the injured nerve, including immune cells (mainly *Sema4a*, *Sema4b*, and *Sema4d*) and SCs themselves (mainly *Sema4c*, *Sema4f*, and *Sema4g*), thus facilitating potential SC interactions via Plexin-B1 (fig. S1B).

As suitable antibodies for Plexin-B1 immunostaining in mouse tissue are currently unavailable, we utilized our *Plxnb1* knockout (KO) allele harboring a *lacZ* reporter cassette (*32*) (Fig. 1C). X-galactosidase (X-Gal) staining of sciatic nerve sections revealed expression of *Plxnb1-lacZ* in the nerve bridge at 7 dpi after transection injury (Fig. 1C). Time-course analysis further demonstrated a progressive increase in *Plxnb1-lacZ* expression from 4 to 14 dpi, with peak levels at 7 dpi (Fig. 1D). This expression pattern colocalized with immunofluorescence (IF) for SC marker S100B (Fig. 1D) (*33*). Together, these findings demonstrate that Plexin-B1 is induced in SCs following nerve injury.

### Plexin-B1 deletion disrupts SC migration and longitudinal alignment in the nerve bridge

To elucidate the role of Plexin-B1 in nerve repair, we compared *Plxnb1*^−/−^ mice and littermate controls after sciatic nerve transection. Mice of both genotypes formed a nerve bridge spanning the lesion gap (fig. S1C). We isolated primary SCs from *Plxnb1*^−/−^ and control mice and conducted IF for SC markers S100B, p75^NTR^, glial fibrillary acidic protein (GFAP), and Sox10 [transcription factor essential for SC development (*34*)] to confirm SC identity and purity of our cultures (fig. S2A). Loss of Plexin-B1 did not alter SC proliferation, as measured by IF for Ki67, nor did it affect induction of c-Jun, a transcription factor critical for SC dedifferentiation (*35*), either in vitro (fig. S2, B and C) or in vivo after sciatic nerve injury (fig. S2, D to F). There was also no significant increase in apoptosis at the injury site in *Plxnb1*^−/−^ mice, as indicated by IF for activated caspase-3 (fig. S2G). We next examined SC motility by live-cell imaging of SC cultures, which revealed reduced motility of *Plxnb1^−/−^* SCs compared to wild-type (WT) cells (fig. S3A); *Plxnb1^−/−^* SCs also showed a modest delay of wound closure in scratch wound assays (fig. S3B), likely reflecting a combined effect of lowered migration capacity and altered directionality or SC-SC interactions.

**Fig. 2.**
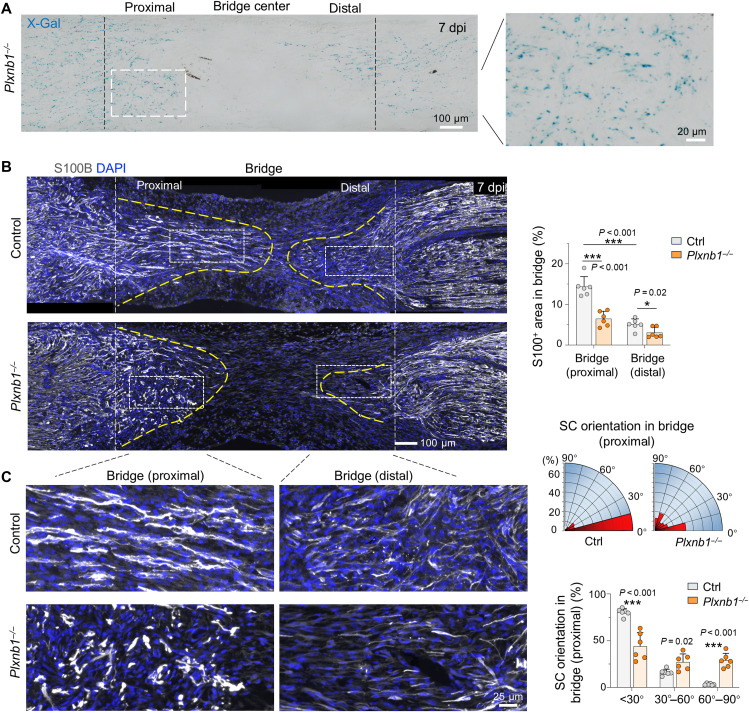
Plexin-B1 deletion disrupts SC alignment in the nerve bridge. (**A**) X-Gal staining of sagittal nerve sections at 7 dpi shows delayed entry of *Plxnb1*-lacZ–positive SCs into the nerve bridge (compare with control condition in Fig. 1C). Boxed area shown at higher magnification to the right. (**B**) Sagittal sections immunostained for S100B reveal reduced SC migration in *Plxnb1*^−/−^ nerves at 7 dpi (*n* = 6 control, *n* = 6 KO; unpaired two-tailed *t* test). *P < 0.05 and ***P < 0.001. (**C**) Zoomed in images from (B) illustrate fusiform, longitudinally aligned SCs in controls, in contrast to stellate, randomly oriented cells in *Plxnb1*^−/−^ mice. Wind rose plots (top right) and corresponding bar graphs (bottom right) show quantification of SC alignment in proximal bridge area relative to the main axis of the nerve. Data are means ± SEM (*n* = 6 control, *n* = 6 KO; unpaired two-tailed *t* test). ***P < 0.001.

We then assessed SC migration and spatial organization within the nerve bridge by X-Gal staining and IF for S100B. As shown in Fig. 1C, by 7 dpi, SCs in control mice had migrated from both proximal and distal nerve stumps and made contact at nerve bridge center, but SCs in *Plxnb1^−/−^* mice exhibited delayed migration and had not yet made contact in the bridge center (Fig. 2, A and B). Quantifications also revealed a greater number of SCs entering from the proximal nerve stump than from distal stump, similar to previous reports (*5*, *8*). This may reflect a stabilization effect of axonal contacts for SC cords in the proximal bridge.

Moreover, *Plxnb1^−/−^* SCs displayed notable defects in alignment and morphology. While control SCs adopted elongated, fusiform morphologies with longitudinal alignment along the nerve axis, more prominent in the proximal bridge, *Plxnb1^−/−^* SCs remained amoeboid or stellate in shape and were frequently misaligned, forming clusters in diagonal orientation relative to the nerve axis (Fig. 2C). Notably, SC alignment in the proximal stump (where residual axon tracks are present) or in the distal stump (where bands of Büngner form) was not significantly affected in *Plxnb1^−/−^* mice (fig. S3C). High-magnification images of c-Jun–stained nuclei in the nerve bridge further illustrated SC misalignment and clustering in mutant mice, in contrast to the orderly arrangement of elongated nuclei in control cohorts (see fig. S2E).

### Plexin-B1 mediates CIL between SCs

**Fig. 3.**
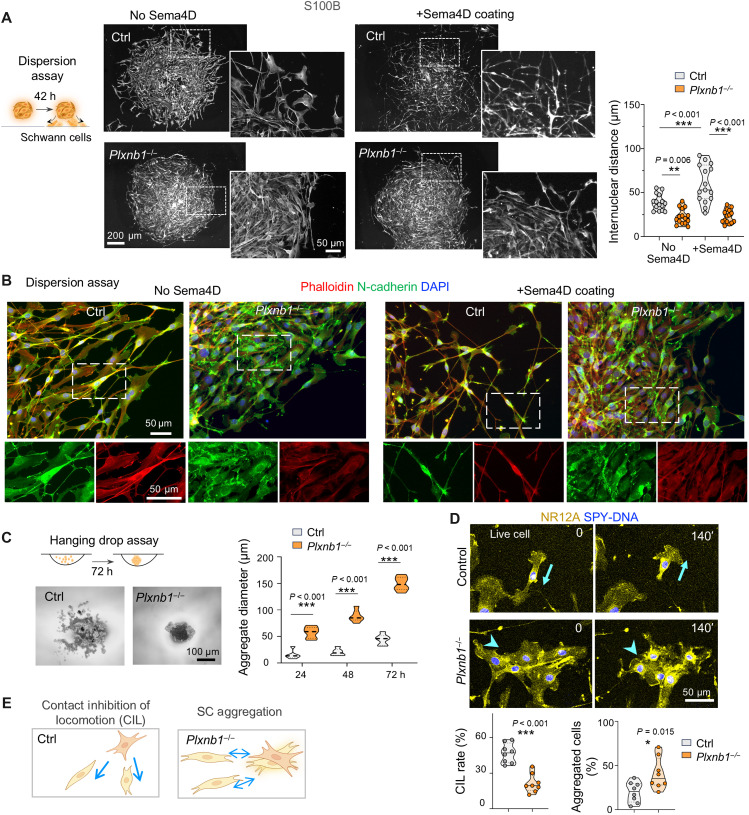
Plexin-B1 loss impairs SC dispersion, motility, and contact inhibition. (**A**) SC sphere dispersion assay showing denser, less dispersed *Plxnb1*^−/−^ spheres at 42 hours (h). Sema4D coating (25 μg/ml) enhanced dispersion of control but not of *Plxnb1^−/−^* SC spheres. Quantification shows internuclear distances (violin plots, median and quartiles; *n* = 15 fields from three cultures; one-way ANOVA with Tukey’s test). **P < 0.01, and ***P < 0.001. (**B**) *Plxnb1^−/−^* SCs displayed reduced F-actin (phalloidin) but increased N-cadherin staining. Sema4D coating alters cytoskeletal organization of dispersing SCs in control but not under *Plxnb1*^−/−^ condition. (**C**) Hanging drop assay showing faster aggregation of *Plxnb1*^−/−^ SCs. Quantifications of growth of aggregates over time shown to the right (*n* = 6 drops per group; two-way ANOVA with Sidak’s test versus control). ***P < 0.001. (**D**) Live-cell imaging of SC collision highlights higher rates of CIL in control SCs than in *Plxnb1*^−/−^ SCs, which tended to form clusters (*n* = 8 fields from two cultures; unpaired two-tailed *t* test). Arrows denote movement direction of control cells, and arrowheads denote aggregation of mutant SCs. (**E**) Schematics of CIL and aggregation behaviors between SCs. Figures in (A), (C), and (E) were created in BioRender [J. Li (2025), https://BioRender.com/m19u012]. *P < 0.05 and ***P < 0.001.

Given that the in vivo injury analyses revealed impaired migration and spatial disorganization as key phenotypes of Plexin-B1 KO during nerve repair, we next investigated cell-cell interactions through three culture assays. First, we performed three-dimensional dispersion assay by seeding aggregated SCs onto laminin-coated dishes, and cell detachment and migration were examined over time (Fig. 3A). At 42 hours postseeding, control SCs had initiated detachment from aggregates and migrated away from center. In contrast, *Plxnb1^−/−^* SCs remained tightly clustered, resulting in high cell density at aggregate core and limited dispersion at periphery (Fig. 3A). This phenotype recapitulates the migration and clustering defects observed in vivo within the nerve bridge. Substrate coating with Sema4D significantly promoted SC dispersion of control but not *Plxnb1^−/−^* cells, illustrating a Sema4D/Plexin-B1–dependent effect (Fig. 3A). Higher-magnification images revealed Sema4D-induced morphological changes of SCs into spindle-like shape. To further explore cytoskeletal and membrane adhesion changes underlying these behaviors, we examined filamentous (F)–actin structures and N-cadherin localization. Phalloidin staining revealed reduced F-actin in *Plxnb1^−/−^* SCs compared to controls, signifying cytoskeletal alteration (Fig. 3B). Conversely, N-cadherin expression was elevated (Fig. 3B), particularly at SC-SC contact sites, consistent with increased adhesion between *Plxnb1^−/−^* SCs (*18*). The Sema4D-induced spindle morphology was again demonstrated by phalloidin staining in control but not mutant SCs.

Second, to further assess cell-cell adhesion, we cultured SCs at high density in hanging drops and monitored aggregation rates over 72 hours. Compared to controls, *Plxnb1^−/−^* SCs formed larger aggregates, and, correspondingly, the number of aggregates was reduced by 48 and 72 hours, indicating enhanced cell-cell adhesiveness (Fig. 3C and fig. S3D).

Third, we used live-cell imaging to directly visualize cell-cell contact behavior. SCs were labeled with membrane dye NR12A and nuclear dye SPY650-DNA and imaged during collisions (Fig. 3D). Control SCs frequently exhibited CIL, a phenomenon where cells halt migration upon collision, repolarize, and then migrate away from each other within minutes (*36*) (movie S1). In contrast, *Plxnb1^−/−^* SCs tended to cluster together upon contact rather than repel each other (Fig. 3D and movie S2). Together, these findings demonstrate that Plexin-B1 regulates multiple aspects of SC behavior, including motility, dispersion, cell-cell adhesion, and CIL (Fig. 3E).

### Membrane remodeling in SCs requires Plexin-B1

**Fig. 4.**
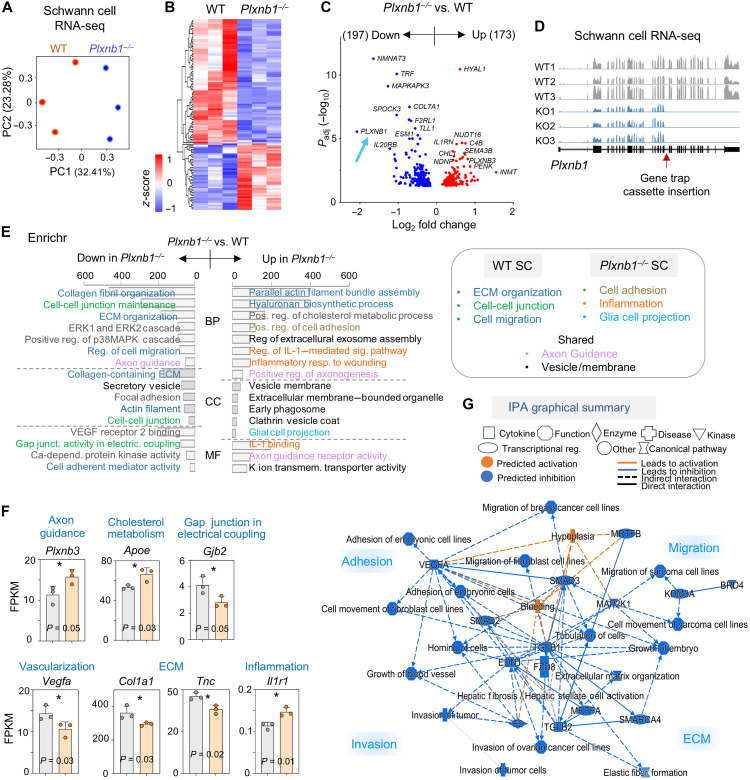
Transcriptomic profiling of *Plxnb1*^−/−^ SCs reveals alterations in membrane, cytoskeletal, and inflammatory gene programs. (**A**) PCA of RNA-seq data shows distinct clustering of WT and *Plxnb1*^−/−^ SC samples. (**B**) Heatmap of differentially expressed genes (DEGs)s from triplicate RNA-seq samples, hierarchically clustered. (**C**) Volcano plot of DEGs of *Plxnb1*^−/−^ versus WT SCs. (**D**) RNA-seq tracks confirm loss of expression of exons downstream of the trap cassette insertion in *Plxnb1*^−/−^ SCs (arrow). (**E**) Enriched up- and down-regulated Gene Ontology terms with color-coded functional themes. Summary to the right. BP, biological process; CC, cellular component; MF, molecular function; ERK, extracellular signal–regulated kinase; MAPK, mitogen-activated protein kinase; VEGF, vascular endothelial growth factor. (**F**) Expression of selected DEGs (FPKM, fragments per kb transcript per million reads; means ± SEM; *n* = 3 per genotype). *P < 0.05. (**G**) Ingenuity Pathway Analysis (IPA) graphical summary of altered pathways in *Plxnb1*^−/−^ versus WT SCs. MAP2K1, MAPK kinase 1; TGFB1, transforming growth factor–β1; MRTFB, myocardin-related transcription factor B; KDM3A, lysine demethylase 3A; BRD4, bromodomain containing 4; EDN1, endothelin 1; FZD8, frizzled class receptor 8; SMARCA4, SWI/SNF related, matrix associated, actin dependent regulator of chromatin, subfamily a, member 4.

To uncover the mechanisms underlying the aberrant behavior of *Plxnb1^−/−^* SCs, we profiled transcriptomics of cultured SCs by RNA-seq. Principal components analysis (PCA) showed clear segregation of WT and *Plxnb1^−/−^* SC samples along PC1, indicating distinct transcriptional changes (Fig. 4A). Differential gene expression analysis revealed 173 up-regulated and 197 down-regulated genes in *Plxnb1^−/−^* SCs (adjusted *P* < 0.05, |log_2_ fold change| > 0.25), with *Plxnb1* itself among the most significantly down-regulated targets (Fig. 4, B to D, and table S1).

Gene Ontology enrichment with Enrichr analysis (*37*) revealed that down-regulated genes were associated with axon guidance, cell migration, ECM organization, and membrane-related processes including cell adhesion, gap junction activity, and cell-cell junctions (Fig. 4, E and F). The up-regulated genes also featured membrane-related functions such as vesicle membrane, clathrin vesicle coating, and extracellular membrane–bounded organelles, as well as biological processes such as glial cell projection, hyaluronan metabolism (a key ECM component), and cholesterol biosynthesis (Fig. 4, E and F). *Plxnb1* deletion also up-regulated genes linked to inflammatory responses, including interleukin-1 (IL-1) signaling and wound-induced inflammation.

Ingenuity Pathway Analysis (IPA) further supported these findings, highlighting a predominant inactivation of pathways related to cell adhesion, migration, invasion, and ECM remodeling in *Plxnb1^−/−^* SCs, with only two activated categories: hypoplasia and bleeding (Fig. 4G; fig. S4, A and B; and table S2). Notably, axon guidance components were enriched among both up-regulated and down-regulated genes, consistent with Plexin-B1 deletion (Fig. 4E and fig. S4, A to C).

**Fig. 5.**
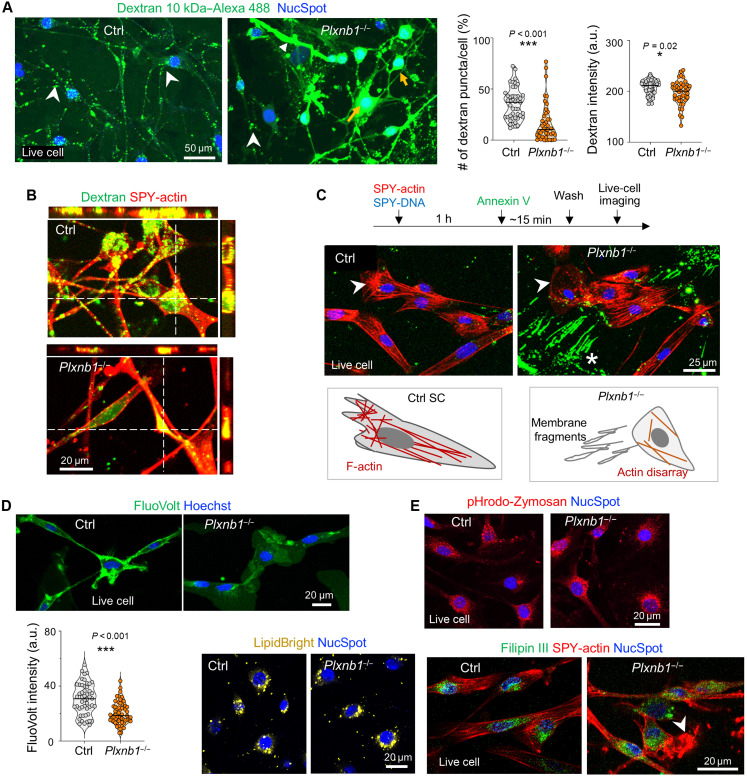
*Plxnb1*^−/−^ SCs display altered membrane properties and cell-cell contact behavior. (**A**) Dextran (10 kDa) uptake assay shows lower levels of endocytic puncta (arrowheads) in *Plxnb1*^−/−^ SCs, but with some cells exhibiting diffuse cytosolic fluorescence (yellow arrows). Quantification in violin plots (median, quartiles; *n* = 45 control and 42 KO cells from three cultures; unpaired two-tailed *t* test). *P < 0.05 and ***P < 0.001. a.u., arbitrary units. (**B**) Confocal images confirm reduced puncta in *Plxnb1*^−/−^ SCs and diffuse dextran labeling in some mutant cells. (**C**) Live-cell imaging reveals annexin V–positive fragments of cell processes (asterisk) and disorganized F-actin (arrowhead) in *Plxnb1*^−/−^ SCs; schematics summarize cytoskeletal defects. (**D**) Live-cell imaging reveals reduced FluoVolt membrane potential signal in *Plxnb1*^−/−^ SCs (violin plots; *n* = 50 control and 57 KO cells from three cultures; unpaired two-tailed *t* test). ***P < 0.001. (**E**) Phagocytosis of pHrodo-Zymosan and lipid staining with LipidBright or Filipin III show comparable patterns between genotypes. Arrowhead indicates disorganized F-actin in mutant SC.

Given that the transcriptomic signatures related to membrane remodeling and actin dynamics, we next performed live-cell imaging to validate predicted functions of Plexin-B1 in mediating membrane-associated processes. We conducted dextran uptake assay, a readout for endocytosis, which showed that *Plxnb1^−/−^* SCs exhibited reduced uptake of fluorescently labeled 10-kDa dextran (visible as puncta in endosomes); in addition, many mutant SCs harbored diffuse cytosolic dextran, suggesting membrane instability allowing dextran entry (Fig. 5, A and B).

To further investigate membrane integrity, we labeled live cells with annexin V, which binds to externalized phosphatidylserine, a hallmark of membrane damage (*38*). While control SCs showed minimal annexin V labeling, *Plxnb1^−/−^* SCs exhibited numerous annexin-labeled membrane structures, illustrating membrane microrupture or shedding of cell fragments (Fig. 5C).

Since cortical actin regulates plasma membrane dynamics, we used SPY-actin to assess F-actin organization in live cells. In contrast to the prominent stress fibers observed in control SCs, *Plxnb1^−/−^* SCs displayed disorganized F-actin architecture (Fig. 5C), suggesting that cytoskeletal dysregulation may contribute to the impaired membrane dynamics and CIL behavior of mutant SCs.

The dynamics of the plasma membrane can affect local membrane electrical charges (*39*). We therefore conducted live-cell imaging with the membrane dye FluoVolt, which changes fluorescence intensity in dependence of membrane electrical charges, with lower signal associated with reduced membrane turnover (*40*, *41*). *Plxnb1^−/−^* SCs exhibited markedly lower FluoVolt signal intensity compared to controls (Fig. 5D), further supporting impaired membrane dynamics of mutant SCs.

Despite the differences in endocytosis and membrane integrity, assays for phagocytosis (pHrodo zymosan), lipid content (LipidBright), and free cholesterol (Filipin III) did not reveal overt differences between *Plxnb1^−/−^* and control SCs (Fig. 5E). Together, these data demonstrate that Plexin-B1 is critical for plasma membrane stability and cytoskeletal dynamics of SCs, features essential for efficient CIL in directing SC alignment in the nerve bridge.

### Axon misalignment and impaired axon regeneration with Plexin-B1 deficiency

**Fig. 6.**
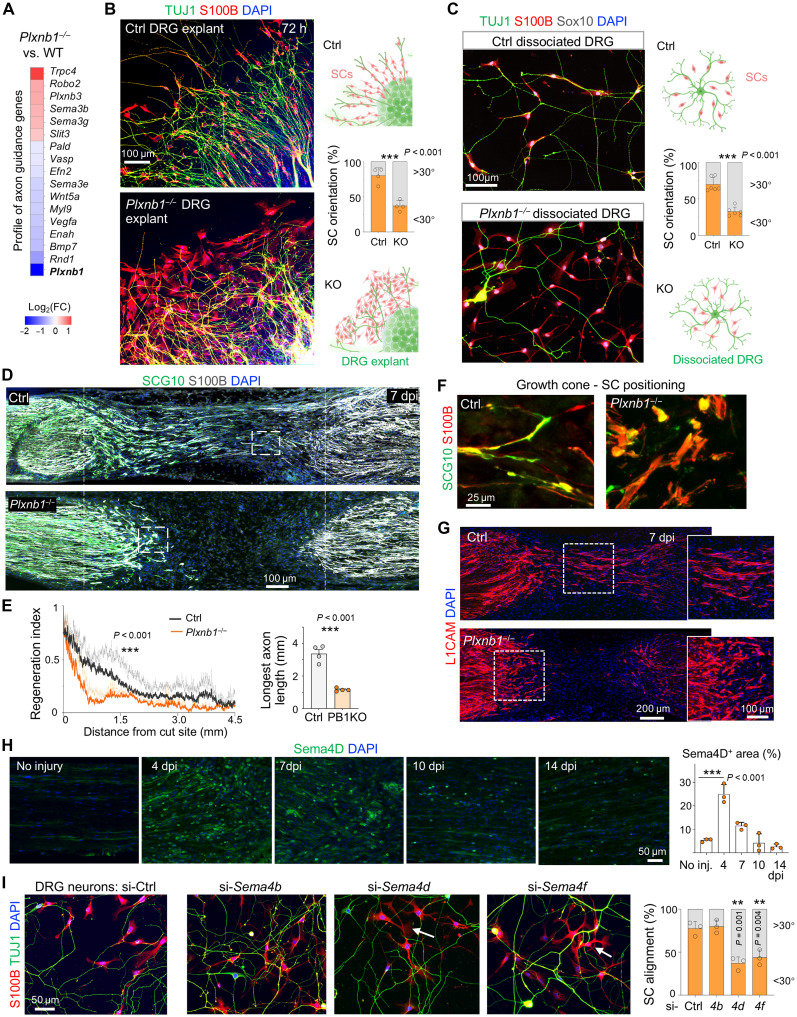
Plexin-B1 deletion results in axon-SC misalignment and delayed axon regeneration after nerve injury. (**A**) Heatmap of top axon guidance-related DEGs of *Plxnb1*^−/−^ SCs, including reduced *Plxnb1* and *Vegfa* expression and induction of *Plxnb3*. FC, fold change. (**B**) DRG explants immunostained for S100B (SCs) and TUJ1 (β3-tubulin; axons) show radial SC-axon alignment in controls versus disorganization in mutants. SC orientation was quantified relative to adjacent axon axes (*n* = 4 explants per genotype; unpaired two-tailed *t* test). ***P < 0.001. (**C**) Dissociated DRG cultures show control SCs aligned with TUJ1^+^ axons versus clustered, misaligned *Plxnb1*^−/−^ SCs (*n* = 6 per group from three cultures; unpaired two-tailed *t* test). ***P < 0.001. (**D**) SCG10 staining at 7 dpi reveals reduced axon regeneration in *Plxnb1*^−/−^ versus control nerves. 4′,6-Diamidino-2-phenylindole (DAPI) for nuclear staining. (**E**) Regeneration index and longest axon length quantified (*n* = 4 per genotype; two-way ANOVA for regeneration index and unpaired two-tailed *t* test for maximal axon length.). ***P < 0.001. (**F**) Higher magnification of regeneration fronts from nerves shown in (D) reveal SC/growth cones alignment in controls versus random orientation in *Plxnb1* mutants. (**G**) L1CAM immunostaining confirms reduced axon regeneration in *Plxnb1*^−/−^ mice. (**H**) Sema4D protein expression in sciatic nerves over 14-day period postinjury (*n* = 3 per time point; one-way ANOVA with Tukey’s test). ***P < 0.001. (**I**) SC-neuron coculture assay after small interfering RNA (siRNA) knockdown of selected Sema4 genes in DRG neurons. SC alignment was imaged and quantified at 30 hours of coculture (*n* = 3 per group from three cultures; one-way ANOVA with Tukey’s test). **P < 0.01. Arrows point to misaligned SCs. Figures in (B) and (C) were created in BioRender [J. Li (2025), https://BioRender.com/m19u012].

As the differentially expressed genes (DEGs) of *Plxnb1^−/−^* SCs featured the axon guidance Gene Ontology (Fig. 6A), we investigated how Plexin-B1 deletion would affect SC-axon interactions. We first examined adult dorsal root ganglion (DRG) explant cultures containing both DRG neurons and SCs. At 72 hours after plating, SCs in control explants had aligned along DRG axons, with majority oriented within 30° of axonal trajectories. In contrast, SCs in *Plxnb1^−/−^* explants were disorganized and aggregated together, lacking directional alignment along axons (Fig. 6B), reminiscent of the clustering phenotypes observed in prior assays. Notably, axons in *Plxnb1^−/−^* DRG explants also appeared disoriented, crisscrossing with irregular trajectories.

To further evaluate axon-SC alignment, we analyzed cultures of dissociated DRGs. Consistent with the explant data, control SCs aligned along axons, whereas *Plxnb1^−/−^* SCs displayed random orientations and poor spatial association with axons (Fig. 6C). These results underscore the critical role of Plexin-B1 in facilitating SC alignment along regenerating axons.

Similar axonal phenotypes were also observed in vivo after sciatic nerve injury. In *Plxnb1^−/−^* mice, IF for SCG10, a marker of regenerating sensory axons (*42*), revealed significantly shorter and more misdirected SCG10^+^ axons compared to controls at 7 dpi (Fig. 6, D and E). High-resolution imaging of the regenerating front showed elongated growth cones in close apposition to fusiform SCs in control mice. In contrast, *Plxnb1^−/−^* mice exhibited enlarged, dysmorphic growth cones surrounded by disorganized, clustered SCs (Fig. 6F). Similarly, IF for L1CAM, a homophilic cell adhesion molecule expressed both in SCs and neurites (*43*), demonstrated delayed and disoriented axon regeneration in *Plxnb1^−/−^* mice (Fig. 6G).

A time-course analysis revealed that nerve bridge formation was initiated in both genotypes at 4 dpi after transection injury, at which time axons had not yet entered the bridge and SCs remained amoeboid (fig. S5A). By 7 dpi, SC migration and alignment defects became evident in *Plxnb1^−/−^* mice and persisted through 10 and 14 dpi, particularly at lesion center (fig. S5A). Infiltration of IBA1^+^ macrophages and platelet-derived growth factor receptor α–positive (PDGFRα^+^) fibroblasts was comparable between genotypes at 4 dpi. These cells entered the nerve bridge ahead of SCs and exhibited random, stellate morphologies, likely due to the absence of SC scaffolds or regenerating axons (fig. S5, B and C).

To identify potential Plexin-B1 ligands involved in SC-axon interaction, we analyzed a single-cell RNA-seq dataset of DRGs after peripheral nerve injury (*44*). Among the class 4 semaphorins, *Sema4b*, *Sema4d*, and *Sema4f* were highly expressed in DRG neurons postinjury, whereas *Plxnb1* was predominantly expressed in SCs (fig. S6A). SCs in injured nerve also express high levels of *Sema4c* (fig. S6A). IF of nerve tissue revealed that Sema4D protein was up-regulated between 4 to 7 dpi and declined by 14 dpi after nerve transection (Fig. 6H). Coimmunostaining at 7 dpi showed that Sema4D was highly expressed in SCG10^+^ regenerating axons and partially overlapped with IBA1^+^ macrophages and CD45^+^ leukocytes, but not with S100B^+^ SCs (fig. S6B). Consistently, in primary cultures, Sema4D was robustly detected in DRG neurons (on both soma and neurites) and at low levels in macrophages but not in SCs (fig. S6C).

To assess the functional role of semaphorins in axon-SC interaction, we performed small interfering RNA (siRNA) knockdown in DRG neurons. Knockdown of *Sema4d* or *Sema4f* in DRG neurons perturbed SC alignment along axons, whereas knockdown of *Sema4b* in DRG neurons had no discernible effect (Fig. 6I). These findings suggest that axonal Sema4D and Sema4F may activate Plexin-B1 to facilitate SC alignment along axons and stabilize SC cords in the proximal bridge. Notably, Sema4F, a membrane-bound semaphorin, is also expressed at lower levels by SCs themselves, which may mediate initial SC-SC alignment via CIL. In the context of neurofibromatosis, Sema4F expression in SCs is also critical for axon-SC contacts to repress SC proliferation (*30*).

### Heightened inflammation and compromised functional recovery in Plexin-B1–deficient mice

**Fig. 7.**
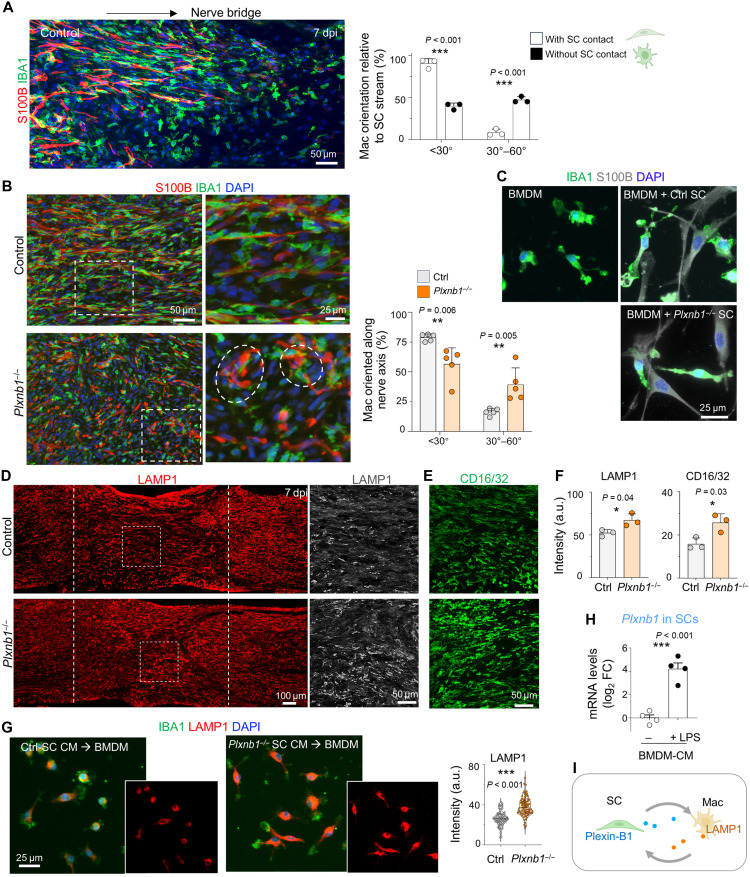
Spatial disarray in the nerve bridge of *Plxnb1*^−/−^ mice is linked to heightened inflammation. (**A**) In control nerves at 7 dpi, IBA1^+^ macrophages arrive before S100B^+^ SCs, appearing stellate without SC contacts, but fusiform and aligned when in contact (*n* = 3 per group; unpaired two-tailed *t* test). ***P < 0.001. (**B**) At 7 dpi, nerve bridges of control mice show both macrophages and SCs aligned longitudinally, whereas bridges in *Plxnb1*^−/−^ mice contain stellate, randomly oriented, clustered cells (*n* = 5 per genotype; unpaired two-tailed *t* test). **P < 0.01. (**C**) Bone marrow–derived macrophages (BMDMs) cocultured with control or *Plxnb1*^−/−^ SCs extend processes similarly alongside elongated SCs. (**D** to **F**) Elevated LAMP1 and CD16/32 signals in *Plxnb1*^−/−^ nerve bridges at 7 dpi and increased clustering/disarray (*n* = 3 per genotype; unpaired two-tailed *t* test). *P < 0.05 (**G**) BMDMs exposed to conditioned medium (CM) from *Plxnb1*^−/−^ SCs show higher LAMP1 levels (violin plots; *n* = 85 Ctrl versus 83 KO for LAMP1; unpaired two-tailed *t* test). ***P < 0.001. (**H**) Quantitative reverse transcription polymerase chain reaction (qRT-PCR) shows *Plxnb1* induction in SCs treated with CM from lipopolysaccharide (LPS)–stimulated BMDMs, but not by CM from unstimulated BMDMs. ***P < 0.001. (**I**) Diagram of reciprocal SC-macrophage interactions: Inflammatory macrophages induce *Plxnb1* in SCs and SC-secreted factors reduce macrophage lysosomal activation. Figures in (A) and (I) were created in BioRender [J. Li (2025), https://BioRender.com/m19u012].

Following nerve injury, blood-borne macrophages rapidly infiltrate the damaged nerve and arrive in the nerve bridge ahead of SCs (*19*). In control mice, by 7 dpi, IBA1^+^ macrophages in the proximal region of the bridge were aligned with SCs, both adopting fusiform shapes along the nerve axis. In contrast, macrophages in the central bridge region that were devoid of axonal and SC contacts exhibited ameboid or stellate shapes with random orientation (Fig. 7A), illustrating the instructive role of axons and SCs in directing immune cell morphology and spatial patterning. In *Plxnb1^−/−^* mice with SC-axon misalignment, macrophages remained disorganized and maintained an ameboid morphology even in the proximal bridge (Fig. 7B).

To determine whether Plexin-B1 influences SC-macrophage interactions directly, we cocultured bone marrow–derived macrophages (BMDMs) with either control or *Plxnb1^−/−^* SCs. While BMDMs extended processes upon contact with fusiform SCs, no longitudinal alignment occurred, regardless of control or *Plxnb1^−/−^* SCs (Fig. 7C). This illustrates the importance of axons in directing glial alignment.

Transcriptomic analysis of *Plxnb1^−/−^* SCs revealed enrichment of inflammation-associated gene ontologies (Fig. 4E and fig. S7A). In vivo, the spatial disarray of SCs and axons in *mutant* mice was associated with heightened inflammation, evidenced by increased expression of LAMP1 (a lysosomal/autophagy marker up-regulated in activated macrophages) and CD16/32 (a proinflammatory marker) at 7 dpi (Fig. 7, D to F). Conversely, the expression of CD206 (an anti-inflammatory, prorepair marker) was decreased, particularly at the regenerating front, suggesting a less supportive regenerative environment (fig. S7, B and C). Furthermore, CD206^+^ macrophages were also disorganized in *Plxnb1^−/−^* mice, in contrast to the aligned arrangement in WT mice (fig. S7C). Notably, immune cell recruitment into the bridge, shown by pan-leukocyte marker CD45, was comparable between genotypes (fig. S7C).

To study whether SCs and macrophages communicate via soluble factors, we performed reciprocal conditioned medium (CM) experiments. BMDMs exposed to CM from *Plxnb1^−/−^* SCs exhibited a significant increase in LAMP1 expression relative to those treated with CM grom control SCs, consistent with in vivo findings (Fig. 7G). Plexin-B1 deletion did not alter LAMP1 expression within SCs themselves (fig. S7D). Conversely, SCs exposed to CM from lipopolysaccharide (LPS)–stimulated BMDMs showed a marked up-regulation of *Plxnb1* mRNA (Fig. 7H), indicating bidirectional signaling between SCs and macrophages via soluble factors (Fig. 7I).

**Fig. 8.**
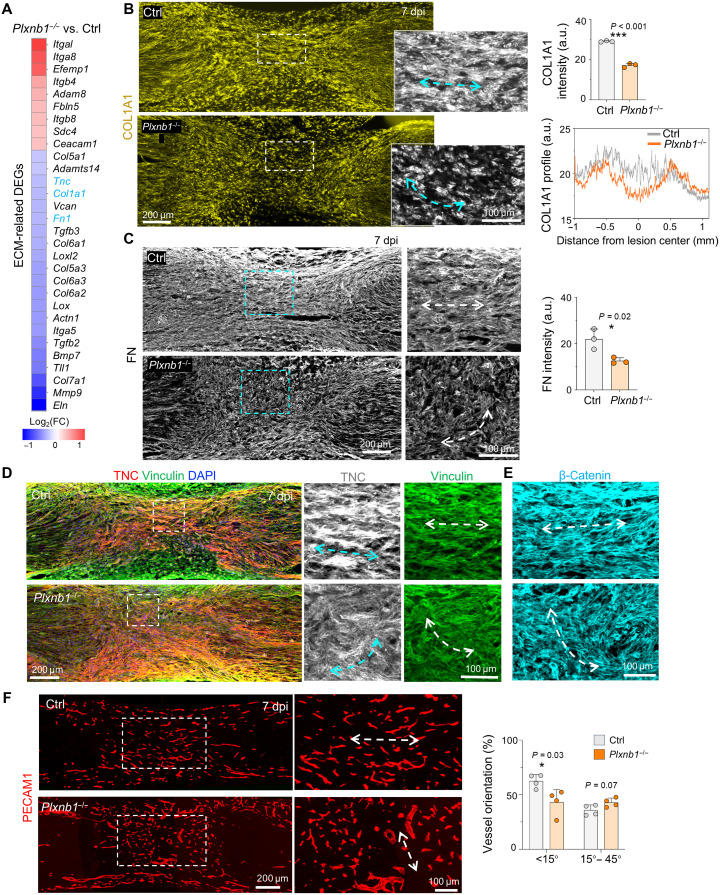
ECM and vascular disorganization at nerve injury site in *Plxnb1*^−/−^ mice. (**A**) Heatmap of ECM-related DEGs of *Plxnb1*^−/−^ SCs, from RNA-seq analysis. (**B** to **E**) Immunostaining at 7 dpi shows reduced level and disorganization of COL1A1 (B), FN (C), TNC and vinculin (D), and β-catenin (E) in *Plxnb1*^−/−^ nerve bridges (*n* = 3 per genotype; unpaired two-tailed *t* test). *P < 0.05 and ***P < 0.001. (**F**) Platelet endothelial cell adhesion molecule 1–positive (PECAM1^+^) vasculature at 7 dpi shows disarray and engorgement in *Plxnb1*^−/−^ nerves compared with controls (*n* = 4 per genotype; unpaired two-tailed *t* test). *P < 0.05. Arrows denote main orientation axes.

RNA-seq analysis also revealed reduced expression of ECM genes including *Col1a1*, *Fn1*, and *Tnc* in *Plxnb1^−/−^* SCs (Fig. 8A). Since ECM undergoes marked remodeling during tissue repair, collective cell migration can influence ECM fiber orientation, a process involving cell collision guidance via axon guidance pathways (*45*–*47*). Correspondingly, IF showed lower expression and spatial disarray of ECM in the nerve bridge of *Plxnb1^−/−^* mice at 7 dpi (Fig. 8, B to D). Additional IF staining revealed disorganization of vinculin (a focal adhesion protein) and β-catenin (critical for cell-cell adhesion), along with abnormal vertical orientation of blood vessels in *Plxnb1^−/−^* mice (Fig. 8, D to F). These histological findings illustrate that Plexin-B1 loss results in SC misalignment and heighted inflammation, disrupting regeneration tracks.

Despite critical impairment in injury response, Plexin-B1 was not essential for peripheral nerve development or SC myelination under homeostatic conditions. In uninjured nerves, SC morphology, myelination, and axon patterning appeared normal (fig. S8, A and B). Similarly, myelin breakdown after injury [assessed by myelin basic protein–positive (MBP^+^) debris in distal nerve] and phagocytosis by IBA1^+^ macrophages and S100^+^ SCs were comparable across genotypes (fig. S8B), consistent with intact phagocytic capacity of *Plxnb1^−/−^* SCs (see fig. 5E). Remyelination of regenerating axons was also similar in dissociated DRG cultures stimulated with ascorbic acid (fig. S9A). In vivo at 35 dpi, IF staining for MBP in the distal nerve showed similar overall remyelination patterns, although spatial disarray and slight reduction in MBP signals were observed in the nerve bridge of *Plxnb1^−/−^* mice (fig. S9B).

**Fig. 9.**
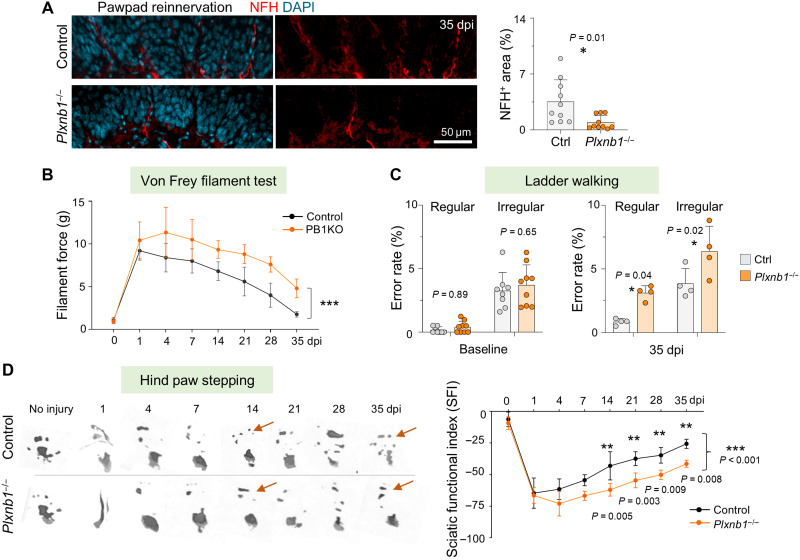
*Plxnb1*^−/−^ mice show delayed functional recovery after sciatic nerve injury. (**A**) NFH immunostaining at 35 dpi reveals reduced reinnervation of the hind paw pad in *Plxnb1*^−/−^ mice (*n* = 10 fields from three mice per genotype; unpaired two-tailed *t* test). (**B**) Von Frey assay shows reduced tactile recovery in *Plxnb1* mutants (*n* = 5 to 7 mice per genotype; two-way ANOVA with Sidak’s test). (**C**) Ladder walking on regular and irregular rungs shows higher error rates at 35 dpi in mutants, with similar baseline performance (*n* = 8 Ctrl and 9 KO at baseline; *n* = 4 per genotype at 35 dpi; two-way ANOVA with Sidak’s test). *P < 0.05. (**D**) Paw print analysis after injury shows impaired recovery in *Plxnb1* mutants. SFI (formula in Materials and Methods) was calculated from prints (*n* = 3 to 7 mice per condition; two-way ANOVA with Sidak’s test). **P < 0.01, and ***P < 0.001.

To further assess the impact of Plexin-B1 deletion on nerve repair, we examined skin reinnervation of the hind paw at 35 dpi. *Plxnb1^−/−^* mice exhibited reduced density of NFH^+^ axon terminals in the epidermis of the paw pad (Fig. 9A). Motosensory functional assessment, including von Frey filament assay (tactile mechanical sensitivity) and ladder walking (coordination) and sciatic functional index (SFI; paw placement during ambulation) measurements revealed significant delays in functional recovery in *Plxnb1^−/−^* mice, starting from 14 dpi and persisting through 35 dpi (Fig. 9, B to D).

### Plexin-B1 conditional KO in SCs disrupts SC-axon alignment and impairs axon regrowth

To confirm the SC-specific function of Plexin-B1 in peripheral nerve regeneration, we generated a floxed *Plxnb1* mouse strain (*48*). For targeted deletion in SCs, we performed intrasciatic injection of adeno-associated virus 9 (AAV9) Cre under the control of an MBP promoter (fig. S10A). To assess Cre specificity for SCs, we also used a Rosa26-LSL-tdTomato reporter strain. Injection of AAV9 MBP-Cre into the sciatic nerve resulted in abundant tdTomato^+^ cells in the nerve bridge, colocalizing predominantly with S100B^+^ SCs, with minimal overlap with PDGFRα^+^ fibroblasts. Quantification revealed over 80% transduction efficiency of SCs in the lesion site (fig. S10, B to D).

**Fig. 10.**
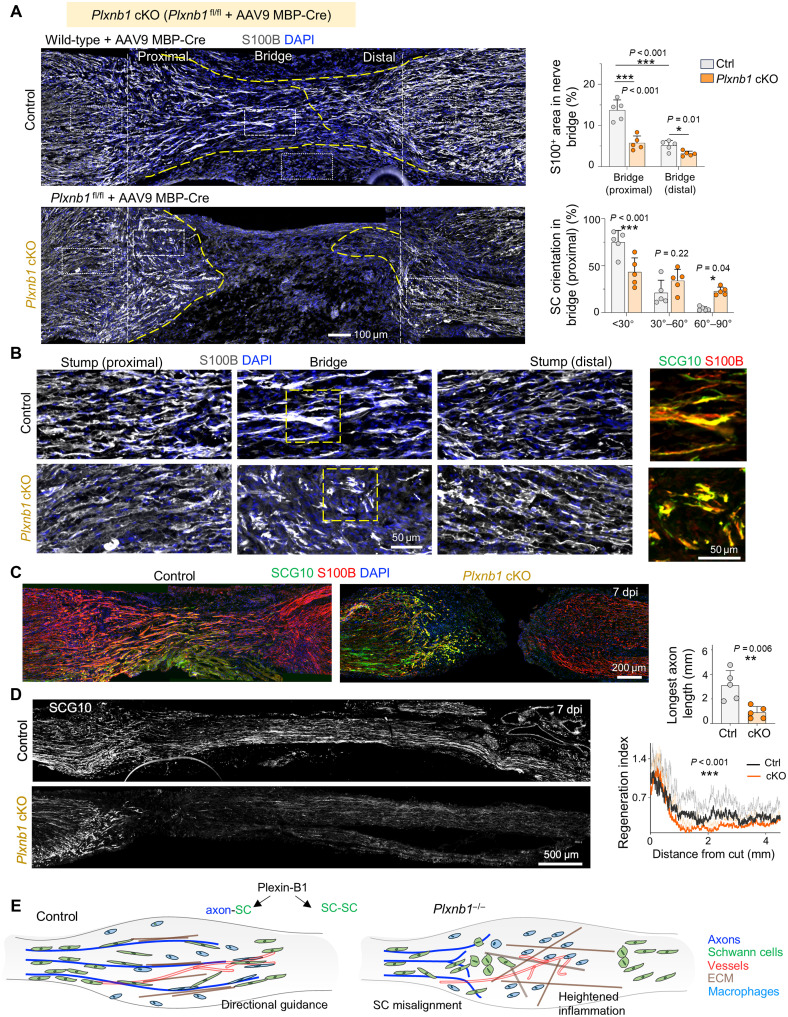
Conditional deletion of Plexin-B1 impairs SC alignment and axon regeneration. (**A**) S100B immunostaining and quantification of sciatic nerves from control or SC-specific *Plxnb1* conditional KO (cKO) mice at 7 dpi after transection. Conditional KO was induced by intrasciatic injection of AAV9 MBP-Cre viral vector at injury. Disorganized SCs are evident in conditional KO nerves. Means ± SEM; *n* = 5 mice per genotype; two-tailed unpaired *t* test. *P < 0.05 and ***P < 0.001. (**B**) High-magnification images of proximal stump, bridge, and distal stump from (A) show altered SC morphology in *Plxnb1* conditional KO nerves. Insets (right) display coimmunostaining for S100B and SCG10, highlighting disorganized SC-axon association in conditional KO nerves, accompanied by growth cone stalling. (**C**) S100B and SCG10 staining show SC and axon disorganization in *Plxnb1* conditional KO nerves. (**D**) SCG10 and S100B IF with quantification reveals reduced axon regeneration in conditional KO mice. Means ± SEM; *n* = 5 per genotype; two-way ANOVA for regeneration index and unpaired two-tailed *t* test for maximal axon length. **P < 0.01, and ***P < 0.001. (**E**) Schematic of Plexin-B1–dependent directional guidance for SC-SC CIL and axon-SC alignment to facilitate nerve regeneration.

This AAV9 MBP-Cre–mediated SC-specific conditional KO of *Plxnb1* recapitulated the phenotypes observed in the constitutive KO, including delayed SC migration and increased clustering of SCs after nerve transection (Fig. 10, A and B). Axon regeneration was also significantly impaired (Fig. 10, C and D), confirming that Plexin-B1 functions cell-autonomously in SCs to coordinate axon-SC alignment and support axonal regrowth following injury.

## DISCUSSION

Peripheral nerves have a remarkable ability to regenerate (*49*), yet recovery after a transection injury often remains slow and incomplete. A central question is how glial cells become oriented in the nerve bridge along the original nerve axis. Here, we demonstrate that SCs require Plexin-B1 for morphological transformation, CIL, and axon-SC alignment during nerve repair (Fig. 10E). This finding establishes a critical role of direct physical contacts for SC polarization and orientation in the nerve bridge.

Axon guidance molecules are key regulators of nerve repair: Netrin/DCC/Unc5H2 signaling promotes SC activation and axonal regrowth (*11*), while Ephrin/EphB signaling directs fibroblast-SC interactions (*50*). Slit repulsive cues mediate CIL between SCs, with Slit2/3 deletion leading to increased aggregation and impaired repulsion (*18*), a phenotype reminiscent of Plexin-B1 KO. This similarity suggests potential convergence between these pathways for CIL. Although Slit binding to Plexin-A has been reported in spinal cord development (*51*, *52*), Slit binding to Plexin-B1 has not been detected in binding assays (*52*). Whether functional cross-talk exists between Slit and Plexin-B1 in nerve repair warrants further investigation. In addition, while Plexins signal through a conserved intracellular Ras–guanosine triphosphatase activating protein domain (*53*), the specific downstream effectors in activated SCs remain to be defined.

Our work underscores that positional guidance for SCs depends not only on soluble cues but also on direct physical contacts. This contact-based principle likely extends to other glial cells in the lesion area, influencing the alignment of vasculature and ECM along the nerve axis. Supporting this model, we recently showed that following nerve injury, Plexin-B2 is up-regulated in macrophages to direct their alignment along axons via CIL (*19*). Thus, different glial cells engage distinct plexins to achieve spatial organization through collision-based mechanisms. This principle also applies to the central nervous system (CNS): After spinal cord injury (SCI), Plexin-B2 in microglia/macrophages and Plexin-B1 in reactive astrocytes orchestrate glial alignment (*26*, *27*); in Alzheimer’s disease, Plexin-B1 organizes astrocyte spacing in periplaque nets (*28*).

Live-cell imaging of *Plxnb1^−/−^* SCs revealed several membrane-associated defects, providing a mechanistic basis for the impaired CIL and axon-SC misalignment. Conceptually, CIL differs from classical cell repulsion: CIL is a specific, directional response upon cell collision to actively turn away and move in a new direction. The main candidate to mediate CIL between SCs is Sema4C, while Sema4D and Sema4F expressed in axons are strong candidates for mediating axon-SC alignment.

Transcriptomic data further indicate that Plexin-B1 deletion dysregulates pathways involved in membrane turnover and vesicle trafficking. These findings extend the known importance of membrane integrity for cell motility and adhesion (*39*) and complement our recent work on mechanoelectrical regulation of the plasma membrane as mediated by Plexin-B2 in glioma cell migration (*41*).

Our study also links spatial disorganization during tissue repair to heightened inflammation. RNA-seq analysis of cultured *Plxnb1^−/−^* SCs revealed up-regulation of inflammatory pathways even without an in vivo injury context. In coculture, macrophages exposed to conditioned media from mutant SCs showed increased LAMP1 expression. Conversely, SCs up-regulate *Plxnb1* expression when exposed to activated macrophage media. Thus, beyond contact-mediated CIL, SCs and macrophages engage in bidirectional communication via secreted factors. This interplay is further shaped by cellular heterogeneity: Most bridge macrophages are monocyte-derived, while those in the proximal stump are resident tissue macrophages (*8*), and SCs themselves adopt distinct states in different bridge zones (*54*).

A lingering question is why the glial responses in CNS and peripheral injury are different. After SCI, microglia/macrophages accumulate in the necrotic core bordered by reactive astrocytes. In contrast, peripheral nerve injury triggers SCs into forming aligned cords that guide both axonal regrowth and macrophage positioning. This difference is reflected in vitro: CNS glia cells (astrocytes and microglia) tend to segregate (*26*), while peripheral nervous system (PNS) glia (SCs and macrophages) intermingle. A key distinction may also lie in axonal dynamics; PNS growth cones are highly motile, whereas CNS growth cones become dystrophic. While axonal contacts are not strictly required for SCs to enter the bridge, they likely stabilize the longitudinal alignment of SC cords and may explain why more SCs populate the proximal versus the distal bridge.

Last, unlike the collective migration of cell cords in the rostral migratory stream, we lack direct evidence for long-range collective migration of entire SC cords in the nerve bridge. A plausible model is that the proximal part of an SC cord stabilizes upon contact with regenerating axons, while new SCs are recruited at the leading edge to extend the cord further into the bridge. Definitive testing of this model will require advanced in vivo live-cell imaging.

In summary, directional guidance is a fundamental principle of tissue morphogenesis and repair. In nerve regeneration, spatial disarray not only disrupts the regenerative architecture but also exacerbates inflammation. Our findings establish Plexin-B1 as a critical regulator of SC spatial organization, revealing how axon guidance pathways are utilized to orchestrate cellular responses in neural regeneration.

## MATERIALS AND METHODS

### Mice

All animal procedures were conducted in accordance with protocols approved by the Institutional Animal Care and Use Committee at the Icahn School of Medicine at Mount Sinai (protocol LA11-00108). *Plxnb1* mutant mice (*Plxnb1*^tm1Matl^) were generated by targeted insertion of a transmembrane-lacZ-neo gene trap cassette into the *Plxnb1* locus (*32*, *55*). Because *Plxnb1* KO mice show no overt phenotypic abnormalities (*56*, *57*) and *Plxnb1*^+/−^ mice display no detectable phenotypes, both WT and *Plxnb1*^+/−^ littermates were used as experimental controls. The *Plxnb1* conditional allele (*Plxnb1*^em1Frdl^, flox) contains loxP sites flanking exons 20 and 21 (*48*). The *Rosa26* LSL-tdTomato reporter strain Ai14 was obtained from the Jackson Laboratory (strain #007914) (*58*). All mouse lines were maintained on a C57BL/6 background.

### Sciatic nerve injury

Mice were anesthetized, and a small skin incision was made at the mid-thigh using a surgical blade. The fascia between the gluteus superficialis and biceps femoris muscles was gently separated to expose the sciatic nerve. For transection injury, the nerve was completely severed at the proximal third of the femur using Vannas spring scissors (FST, #15000-00), while preserving the integrity of surrounding tissues. Skin was closed with two suture clips. For crush injury, the sciatic nerve was compressed for 15 s using an ultrafine hemostatic forceps (FST, 13021-12).

### AAV intrasciatic injection

The AAV2/9 MBP-Cre vector (10^13^ vg/ml; Obio Inc.) was delivered in 3 μl of aliquots using a 33-gauge syringe (Hamilton, #80008), with three injections spaced within a 5-mm region around the injury site. The sciatic nerve was then transected at the central injection site.

### Behavioral assays

Sensory recovery was assessed using the von Frey assay. Hind paws were stimulated with calibrated von Frey filaments (0.004 to 8 g; Aesthesio), each applied five consecutive times. Paw withdrawal or licking was recorded as a positive response. The von Frey threshold was defined as three positive responses out of five stimulations. Baseline sensitivity was measured before injury.

Motor coordination was evaluated using horizontal ladder walking with regular or irregularly spaced 3-mm-diameter rungs. The home cage was placed at the ladder’s end. Mice completed at least six consecutive runs per trial (3 min) while being video recorded. Side and bottom views (mirror and direct) were analyzed to detect foot faults (drag, slip, or fall) and to assess hindlimb coordination.

Hind paw stepping function was analyzed by paw print assessment. Paws were dipped in ink, and mice walked on paper to record prints. The SFI was calculated as: SFI = −38.3 × (*EPL* − *NPL*)/*NPL* + 109.5 × (*ETS* − *NTS*)/*NTS* + 13.3 × (*EIT* − *NIT*)/*NIT* − 8.8, where *E* is the experimental group, *N* is the normal group, *PL* is the print length, *TS* is the total spread, and *IT* is the intermediate toes.

### DRG neuron and SC cocultures

For DRG explant cultures, DRGs were isolated from adult mice, placed in Dulbecco’s modified Eagle’s medium (DMEM)/F12 on ice, washed twice with ice-cold Hanks’ balanced salt solution (HBSS)–Hepes, and incubated in 0.6% collagenase (Worthington, #LS004196) for 45 min at 37°C. DRGs were rinsed twice with HBSS-Hepes, and two to three ganglia were plated per well on 14-mm poly-l-lysine– and laminin-coated coverslips in 24-well plates. Wells were filled with 200 μl of 10% Matrigel in culture medium [DMEM high glucose with GlutaMAX (Gibco, #10569-044), 10% fetal bovine serum (FBS), and 1× nonessential amino acids]. After 30 min, once DRGs adhered, medium was replaced with 1 ml of Matrigel-free culture medium. Axons extended radially and SCs migrated outward. At 72 hours, cultures were fixed and processed for IF staining.

For dissociated DRG cultures, after collagenase digestion as above, DRGs were washed twice with HBSS-Hepes, digested with 0.25% trypsin containing deoxyribonuclease I (DNase I) (50 mg/ml; Worthington, #LS002138) for 40 min at 37°C, quenched with culture medium, and triturated ~30 times with a fire-polished glass pipette. Dissociated DRG neurons and SCs were plated on poly-l-lysine/laminin-coated coverslips in 24-well plates, cultured for 72 hours, and fixed for IF.

For siRNA experiments, ~4000 purified DRG neurons were resuspended in 1.5 ml of titration medium (without DNase I) and mixed with 0.5 ml of transfection complex containing 2 μl of DharmaFECT 2.0 (Dharmacon, #T-2002-02) and 2 μl of siRNA (20 μM) prediluted in NeuroCult NB-A medium. siRNAs included ON-TARGETplus SMARTpool reagents against mouse *Sema4b* (#L-040786-01-0005), *Sema4d* (#L-047703-01-0005), *Sema4f* (#L-059095-01-0005), or a nontargeting control (#D-001810-10-05) (Horizon). Cells were plated on poly-l-ornithine/laminin-coated plates and maintained under standard culture conditions. Medium was replaced 16 to 18 hours posttransfection. At 48 hours, a subset of cultures was collected for RNA extraction and quantitative reverse transcription polymerase chain reaction (qRT-PCR) validation. For axon-SC interaction assays, 6000 purified WT SCs were added per well at 48 hours posttransfection, cocultured for 30 hours, and fixed for immunostaining.

### SC cultures

To enrich SCs from dissociated DRG cultures (see above), cells were maintained in SC medium containing 2 μM forskolin (Selleck, #S2249), 10 nM neuregulin (PeproTech, #100-03), 10% FBS, 1% nonessential amino acids, and 4% ascorbic acid in DMEM high glucose with GlutaMAX (Gibco, #10569-044) (*59*). Fibroblasts were removed by magnetic cell separation using anti-Thy1 antibody (Miltenyi, #130-101-967), following established protocols (*60*). Medium was replaced every 2 days, and cells were passaged at ~80% confluence. Cells from passages 3 to 5 (purity > 80%) were used for experiments. SCs were cryopreserved in freezing medium containing 50% complete SC medium, 40% FBS, and 10% dimethyl sulfoxide.

### SC/DRG coculture for myelination studies

Purified SCs (~5 × 10^4^) were seeded onto purified DRG neurons isolated from WT mice as described (*61*). Cultures were maintained for 4 days in a 1:1 mixture of SC and DRG media. Medium was then replaced with DMEM containing l-ascorbic acid (50 μg/ml; Sigma-Aldrich, #A0278), nerve growth factor (50 ng/ml), and 15% FBS to induce myelination. Cultures were fed with fresh induction medium every 2 days for 2 weeks. Myelination was assessed by immunostaining for MBP (myelin) and TUJ1 (β3-tubulin; axons).

### Coculture of macrophages and SCs

BMDMs were prepared as described (*19*). BMDMs (4 × 10^4^) and SCs (10^4^) were seeded onto poly-l-ornithine–coated (Sigma-Aldrich) glass coverslips in 24-well plates and cocultured for 24 hours.

### Sphere dispersion essay

SCs (2 × 10^4^ per well) were seeded into ultralow-attachment U-bottom 96-well plates (Corning, #7007). After 4 days, a single sphere formed in each well. Spheres were transferred to laminin-coated (10 μg/ml; Invitrogen) glass chamber slides (Falcon, #354118) and cultured for 42 hours. Cultures were fixed with 3.7% formaldehyde/phosphate-buffered saline (PBS) and processed for IF staining. Nuclear distances were quantified using ImageJ.

### Hanging drop aggregation assay

SCs (10^4^) were seeded in 10-μl droplets on the inverted lid of a 6-cm tissue culture dish. The lid was placed over the corresponding dish containing 5 ml of PBS to maintain humidity during incubation. Images of hanging drops were taken every 24 hours using a stereomicroscope, and sphere number and diameter were quantified using ImageJ.

### Scratch assay

SCs (10^6^ per well) were seeded in six-well plates and grown to confluence. A scratch was made across the center of each well using a 200-μl micropipette tip (*t* = 0 hours). Wound closure was monitored by imaging the scratch area at 12 and 16 hours postscratch.

### SCs cultured on Sema4D-coated substrates

For Sema4D coating, Sema4D-Fc (R&D Systems) at 25 μg/ml was mixed with 1% laminin in PBS, and culture dishes were incubated for 2 hours at 37°C. SCs (10^4^ per well) were seeded in 8-well chamber slides and cultured for 2 days. Cells were fixed and stained by IF for S100B and N-cadherin, and for F-actin using phalloidin–Alexa Fluor 568.

### IF staining

Mice were euthanized by CO_2_ inhalation, and sciatic nerves were dissected and fixed in 4% paraformaldehyde/PBS for 1 hour at 4°C. Tissues were cryoprotected in 12.5% and then 25% sucrose/PBS overnight each, embedded in OCT compound (Tissue-Tek, Thermo Fisher Scientific), and sectioned sagittally at 10 μm in thickness on a cryostat. Sections were collected on SuperFrost^+^ slides (VWR) and stored at −20°C.

For IF, sections were washed three times in PBS and blocked in 5% normal donkey serum (Jackson ImmunoResearch) with 0.3% Triton X-100 (Acros Organics) in PBS for 1 hour at room temperature. Primary antibodies (listed below) were diluted in antibody buffer with 1% bovine serum albumin (Fisher Bioreagents) and 0.3% Triton X-100 in PBS and applied overnight at 4°C. The following day, sections were incubated with fluorophore-conjugated secondary antibodies (Alexa Fluor 488, Alexa Fluor 594, or Alexa Fluor 647; 1:300; Jackson ImmunoResearch) and counterstained with 4′,6-diamidino-2-phenylindole (DAPI; 1:1000; Invitrogen). Slides were mounted with Fluoromount-G (Southern Biotech). Images were acquired on a Zeiss Axioscope widefield fluorescence microscope (Zen Blue v3.6) or a Zeiss LSM780 confocal microscope (Zen Black v8). Primary antibodies for IF are as follows: anti-Col1a1 (host: rabbit, Invitrogen, PA5-29569; 1:200), anti-Iba1 (host: goat, NovusNB, 100-1028; 1:125), anti-Ki67 (host: rabbit, Abcam, ab15580; 1:150), anti-MBP (host: rabbit, Cell Signaling Technology, 78896; 1:50), anti-PDGFRα (host: goat, R&D Systems, AF1062; 1:50), anti-Pecam1/CD31 (host: rat, BD Pharmingen, 553370; 1:250), anti-Reticulin (host: rat, Abcam, ab51824; 1:500), anti-S100B (host: chicken, Aves Labs, S100B; 1:500), anti-SCG10 (host: rabbit, Novus, NBP1-49461; 1:1000), anti-Tuj1 (host: mouse, BioLegend, 801202; 1:1000), anti-c-Jun (host: rabbit, Cell Signaling Technology, 9165; 1:200), anti–phalloidin–Alexa Fluor 568 (Invitrogen, A12380; 1:100), anti-Sox10 (host: goat, R&D Systems, AF2864; 1:50), anti-LAMP1 (host: rat, Developmental Studies Hybridoma Bank, clone1D4B; 1:200), anti-L1CAM (host: goat, R&D Systems, AF277; 1:50), anti-CD16/32 (host: rat, BD Pharmingen, 553142; 1:200), anti-CD45 (host: rat, BD Pharmingen, 550539; 1:200), anti-CD206 (host: goat, R&D Systems, AF2535; 1:200), anti-FN (host: rabbit, EMD Millipore, AB2033; 1:400), anti-TNC (host: rabbit, Millipore, AB19011; 1:100), anti-vinculin (host: mouse, Thermo Fisher Scientific, NB6001293; 1:400), anti–β-catenin (host: mouse, BD Biosciences, 610153; 1:300), anti-NFH (host: chicken, EMD Millipore, AB5539; 1:1000), anti-GFAP (host: mouse, EMD Millipore, MAB360; 1:500), anti–active caspase 3 (host: rabbit, R&D Systems, AF835; 1:200), anti-sema4d (host: rat, Invitrogen, AB1603189; 1:100), anti-CD45 (host: mouse, BD Pharmingen, 610266; 1:200), and anti-p75 (host: rabbit, Millipore, AB1554; 1:200).

### Determination of regeneration index

Merged images of sciatic nerves were generated in Photoshop CC 2019, and SCG10 fluorescence intensity was measured in ImageJ. A rectangular region of interest centered on the lesion site was analyzed using the “plot profile” function, with distance on the *x* axis and mean pixel intensity on the *y* axis. Peak SCG10 intensity was used for normalization, and minimal intensity was used for background subtraction. Maximal axonal length was defined as the most distal point where SCG10 intensity exceeded background.

### X-Gal staining of sciatic nerves

*lacZ* reporter expression was detected in sciatic nerve sections by X-Gal staining. The staining solution contained X-Gal (1 mg/ml; dissolved in dimethylformamide) in buffer containing 0.02% IGEPAL, 0.01% sodium deoxycholate, 5 mM potassium ferricyanide, 5 mM potassium ferrocyanide, 2 mM MgCl_2_ in 0.1 M PBS (pH 7.3). Nerve sections or whole-mount preparations were incubated overnight at 37°C and then imaged using a Zeiss Axioscope (sections) or Nikon (whole mounts) microscope.

### Live-cell imaging

#### Dextran uptake

SCs were incubated at 37°C for 1 hour with NucSpot Live 650 (1:1000; Biotium), SPY555-actin (1:1000; Cytoskeleton), and then with Alexa Fluor 488–dextran (5 mg/ml; molecular weight, 10,000; Invitrogen) for 40 min. Cells were washed with PBS and imaged live on a Zeiss LSM 710 confocal microscope.

#### pHrodo zymosan phagocytosis

SCs were incubated at 37°C for 1 hour with pHrodo (1 mg/ml; Invitrogen) and NucSpot Live 650 (1:1000; Biotium), washed, and imaged live on a Zeiss LSM 710.

#### Membrane potential

FluoVolt membrane dye (1:1000; Thermo Fisher Scientific) with PowerLoad supplement (1:100) in neural stem cell medium was applied for 30 min at 37°C, washed twice, and imaged 15 min later (488-nm excitation) on a Zeiss LSM 710.

#### Cholesterol detection

SCs were preincubated with NucSpot Live 650 (1:1000) for 1 hour at 37°C, then stained with Filipin III (1:200) for 45 min, washed, and imaged live.

#### Lipid droplets

SCs were incubated for 4 hours at 37°C with LipiBright (1:1000; Cytoskeleton) and NucSpot Live 650 (1:1000) in serum-free medium, washed, and imaged live.

#### Annexin V

SCs were incubated for 1 hour at 37°C with NucSpot Live 650 (1:1000) and SPY555-actin (1:1000), followed by Alexa Fluor 488-annexin V (1:40; Thermo Fisher Scientific) for 15 min, washed, and imaged live.

#### Time-lapse nuclear tracking

Control and *Plxnb1^−/−^* SCs were stained with NucSpot DNA 488 (1:1000; Biotium) for 10 min, plated on laminin- or laminin/Sema4D (25 μg/ml)–coated glass-bottom dishes (Cellvis), and imaged for 90 min (1 frame/5 min) using a Leica DMi8 live-cell system. Nuclear movement was quantified using the TrackMate plugin in Fiji (*62*).

### Quantitative reverse transcription polymerase chain reaction

BMDMs were stimulated with LPS (100 ng/ml) for 24 hours. CM with or without LPS was collected and applied to SC cultures for 24 hours. Total RNA was extracted using the RNeasy Plus Maxi Kit (QIAGEN), reverse-transcribed with SuperScript III (Invitrogen), and analyzed by qRT-PCR on an ABI Prism 7900HT system using SYBR Green Master Mix (QuantaBio).

*Hprt1* was used as the housekeeping gene for normalization, and relative gene expression was analyzed using the ΔΔ*C*_t_ method. Primers used are as follows: *Plxnb1*, TCCCCGTTACAAACAGATGG (forward) and TAGAGTTCATGCAGAGCCAC (reverse); *Hprt1*, CCCCAAAATGGTTAAGGTTGC (forward) and AACAAAGTCTGGCCTGTATCC (reverse); *Sema4b*, AGTGTCAAGGAACTCTGCAAG (forward) and GGTGTTCACTGTGTTTGGTTG (reverse); *Sema4d*, GGGAAAAGTGAAGATGGCAAAG (forward) and CTGTGGGAAGAGTTTCGAGAG (reverse); *Sema4f*, GAAATGTCCTTTTGAGCCAGC (forward) and CTGTTCGAATCCAGTCCTCAG (reverse).

### RNA-seq analysis

Total RNA from WT and *Plxnb1^−/−^* SCs was isolated using the RNeasy Micro Kit (QIAGEN) with on-column DNase digestion. RNA quality and concentration were assessed using an Agilent Bioanalyzer (RNA 6000 Pico Kit, 5067-1513). Libraries were sequenced on an Illumina NovaSeq X (Psomagen). FASTQ preprocessing, quality control, and alignment to the mouse mm10 genome were performed using the NGS-Data-Charmer pipeline (https://github.com/shenlab-sinai/NGS-Data-Charmer). Adapters were trimmed with Trim-Galore v0.6.5 (*63*), reads were aligned with Bowtie2 v2.4.1 (*64*), duplicates were removed with SAMtools v1.10 (*65*), and gene counts were generated with FeatureCounts (GENCODE vM25 annotation). Genes detected in fewer than five samples with <2 reads were excluded before differential expression analysis with DESeq2 (*66*).

### Bioinformatics

Heatmaps and volcano plots were generated using FLASKi (*67*) and GraphPad Prism 10. Pathway enrichment was performed with the IPA QIAGEN Knowledge Base (*68*) and Enrichr (*69*). IPA was also used for graphical summaries and subnetwork analyses. For Gene Ontology and Kyoto Encyclopedia of Genes and Genomes analysis, DEG lists were uploaded to Enrichr (https://maayanlab.cloud/Enrichr/) (*70*), and enrichment was reported as the combined score (log-transformed *P* value × *z*-score) (*69*). Single-cell expression dynamics of *Plxnb1* after sciatic nerve injury were obtained from the iNSAT platform (https://cdb-rshiny.med.umich.edu/Giger_iSNAT/) (*31*).

### Statistical analyses

For two-group comparisons, unpaired two-sided *t* tests were used. For datasets with more than two groups, one-way analysis of variance (ANOVA) with Tukey’s or Dunnett’s multiple comparisons tests was applied. For repeated-measures data, two-way repeated-measures ANOVA with Bonferroni or Šidák’s post hoc tests was performed. Analyses were conducted in GraphPad Prism 10 using New England Journal of Medicine-style *P* value reporting. Significance was set at *P < 0.05, **P < 0.01, and ***P < 0.001, with values *P* < 0.001 reported as “*P* < 0.001.”
