# Supplementary material for: Directional guidance to orient Schwann cell alignment in nerve regeneration requires Plexin-B1
Source: Sci Adv. 2026 Apr 24;12(17):eadw4136. doi: 10.1126/sciadv.adw4136 (PMC13108569; doi:10.1126/sciadv.adw4136)
Supplement: Supplementary file 1 — Figs. S1 to S10 Legends for tables S1 and S2 Legends for movies S1 and S2 [file sciadv.adw4136_sm.pdf]

Science Advances
AAAS

Supplementary Materials for

Directional guidance to orient Schwann cell alignment in nerve regeneration requires Plexin-B1

Jiaxi Li *et al.*

Corresponding author: Roland H. Friedel, roland.friedel@mssm.edu;
Hongyan Zou, hongyan.zou@mssm.edu

Sci. Adv. **12**, eadw4136 (2026)
DOI: 10.1126/sciadv.adw4136

The PDF file includes:

Figs. S1 to S10
Legends for tables S1 and S2
Legends for movies S1 and S2

Other Supplementary Material for this manuscript includes the following:

Tables S1 and S2
Movies S1 and S2

Fig. S1

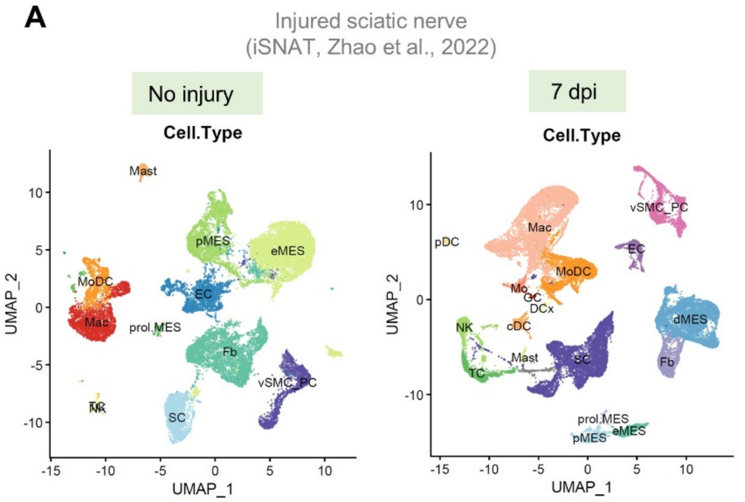

Abbrev.	Cell type
GC	Granulocytes (incl. neutrophils and eosinophils)
Mo	Monocytes
Mac	Macrophages
MoDC	Monocyte-derived dendritic cells
cDC	Conventional dendritic cells
pDC	Plasmacytoid dendritic cells
DCx	Mature/migrating dendritic cells
Mast	mast cells
NK	Natural killer cells
TC	T cells
eMES	Endoneurial mesenchymal cells
pMES	Perineurial mesenchymal cells
dMES	Differentiating mesenchymal cells
prol.MES	Proliferating mesenchymal cells
Fb	Fibroblasts
SC	Schwann cells
EC	Endothelial cells
vSMC_PC	Vascular smooth muscle cells and Pericytes
NA	Unannotated

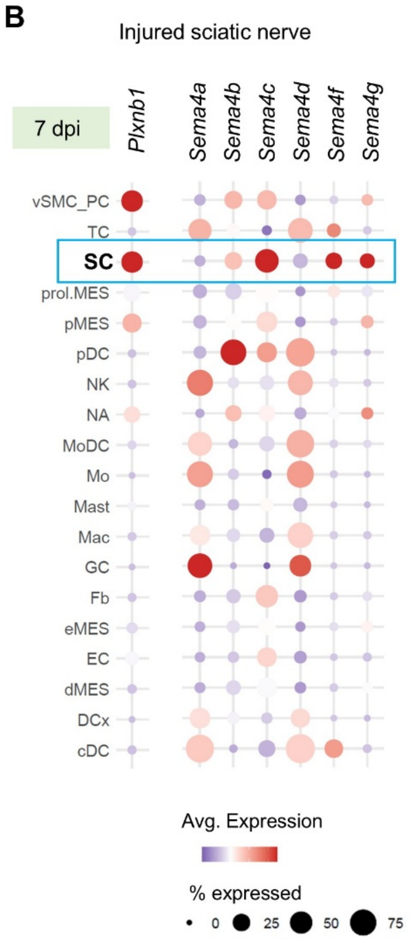

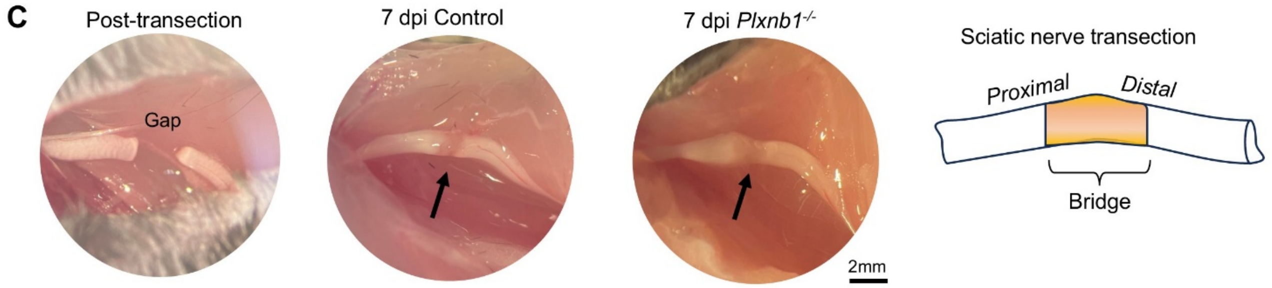

Fig. S1. Plexin-B1 is induced in Schwann cells after sciatic nerve injury.

(**A**) Cell types in the sciatic nerve injury iSNAT datasets (Zhao et al (*31*)).

(**B**) Expression of *Plxnb1* and all *Sema4* genes in sciatic nerve cell types, based on iSNAT single-cell RNA-seq data (7 dpi).

(**C**) Left, sciatic nerve immediately after transection, showing a gap between proximal and distal stumps. By 7 dpi, a nerve bridge connecting the stumps formed in both control and *Plxnb1* KO mice.

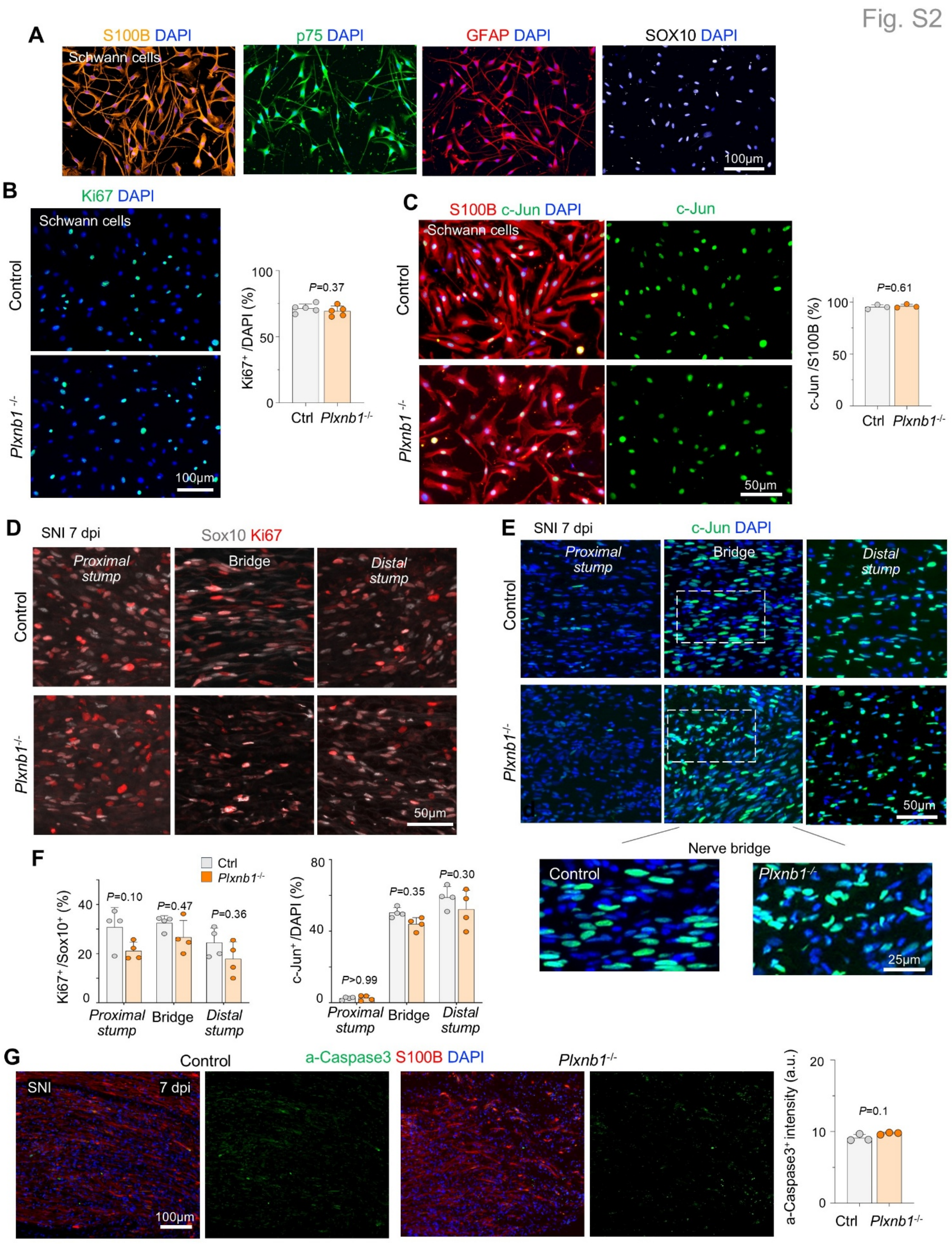

Fig. S2. Plexin-B1 deletion does not affect Schwann cell proliferation and c-Jun induction.
(**A**) Immunostaining of primary SC cultures confirms high purity.

(**B, C**) Primary SCs from control and *Plxnb1*$^{-/-}$ mice show similar Ki67 and c-Jun expression. Mean ± SEM; Ki67, n=5; c-Jun, n=3; three independent cultures; two-tailed unpaired t-tests.

(**D-F**) Sciatic nerve sections at 7 dpi. In proximal and distal stumps, SCs of both genotypes show elongated nuclei and normal c-Jun/Sox10 patterns. In the bridge, control SCs align with the nerve axis, whereas *Plxnb1*$^{-/-}$ SCs show clustered, misoriented nuclei. No significant differences in c-Jun or Ki67 expression across regions. n=4; one-way ANOVA with Tukey's post hoc test.

(**G**) Activated caspase-3 staining shows no increase in apoptosis in *Plxnb1*$^{-/-}$ bridges at 7 dpi. n=3; two-tailed unpaired t-tests.

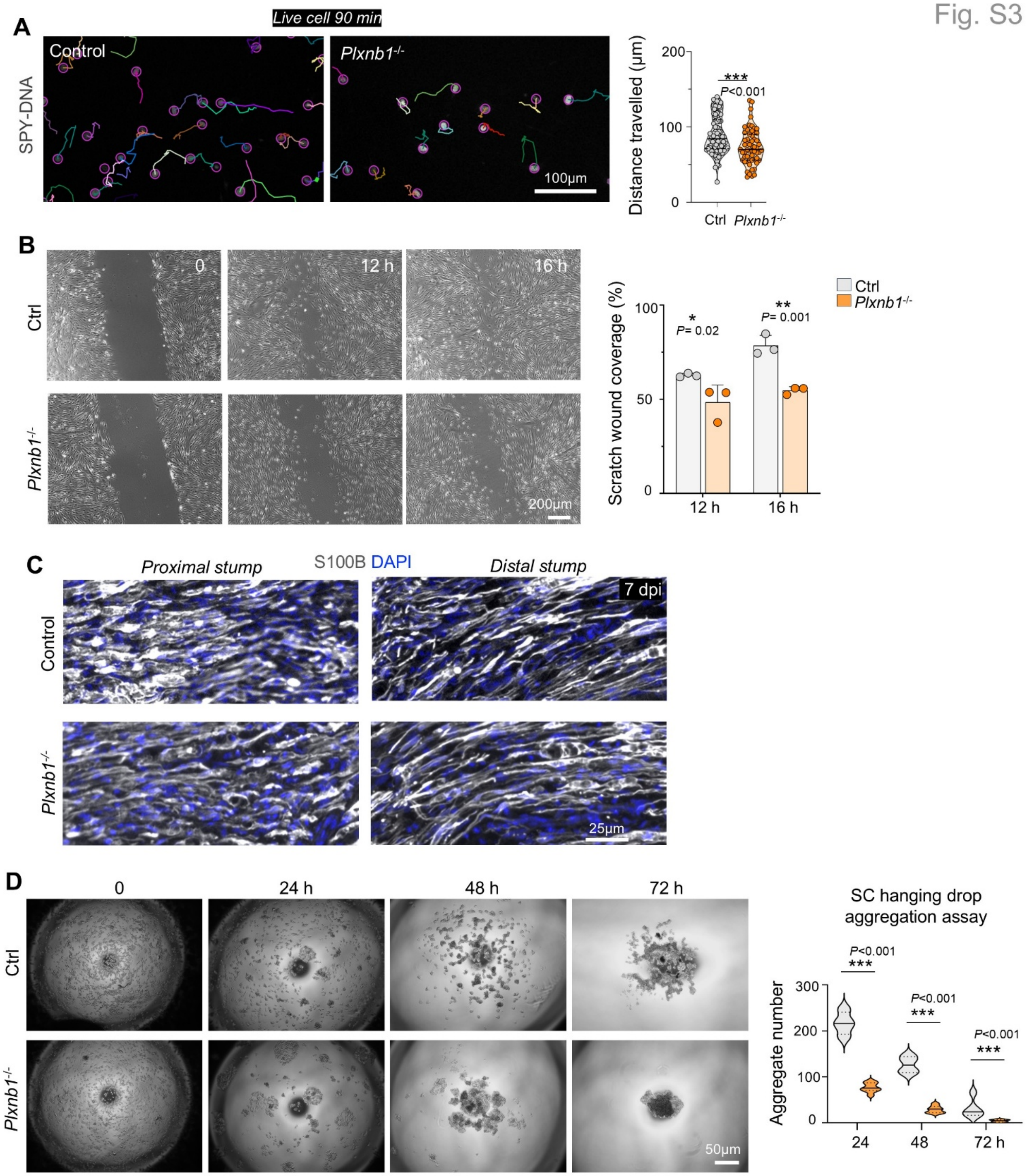

Fig. S3. Plexin-B1-deficient Schwann cells show altered migration and aggregation.

(**A**) Migration distance of cultured SCs over 90 min by live-cell imaging; n=45 (Ctrl) and 42 (*Plxnb1*$^{-/-}$), three independent cultures; two-tailed unpaired t-tests.

(**B**) Scratch wound assay shows reduced closure of scratch area by *Plxnb1*$^{-/-}$ SCs at 12 and 16 h. Mean ± SEM; n=3, three cultures; two-tailed unpaired t-tests.

(**C**) High magnification of S100 immunostaining of proximal and distal stumps (see Fig. 2B) shows no major disruption in SC alignment (compare disrupted alignment in bridge, Fig. 2C).

(**D**) Hanging drop assay shows faster aggregation of floating *Plxnb1*$^{-/-}$ SCs at 24-72 h into large aggregates, reflected by lower overall aggerate numbers. Violin plots show median and quartiles; n=4–8 drops/group; two-way ANOVA with Sidak's test.

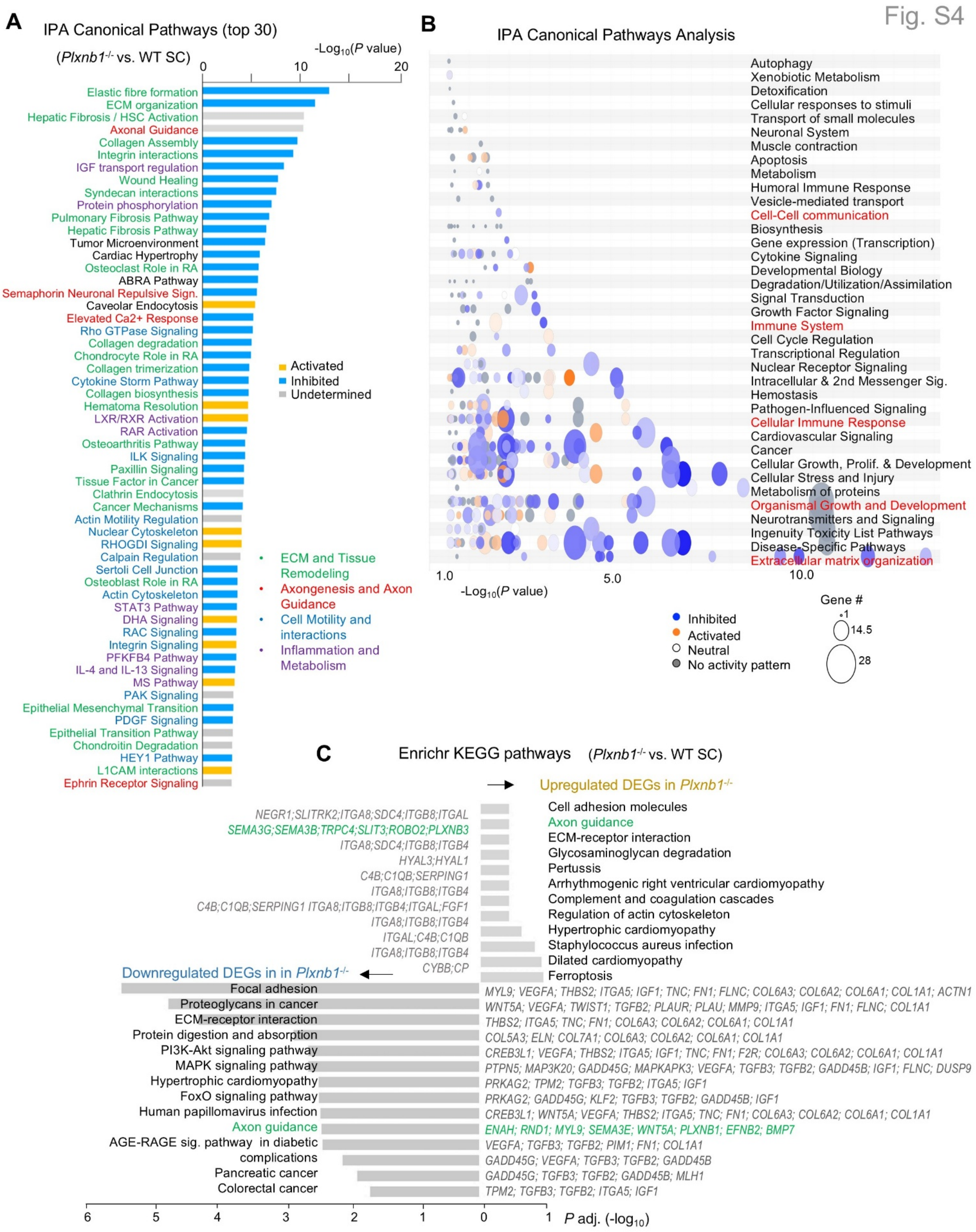

Fig. S4. Transcriptomic changes in *Plxnb1*$^{-/-}$ Schwann cells involve membrane, cytoskeletal, and inflammatory processes.

(**A**) Ingenuity Pathway analysis (IPA) of canonical pathway enrichment of DEGs (*Plxnb1*$^{-/-}$ vs. WT SCs; *P*<0.05; |$\log_2$FC|>0.25).
(**B**) Summary of enriched IPA canonical pathways.
(**C**) Enrichr KEGG pathway enrichment analysis of DEGs; axon guidance genes highlighted in green. *Plxnb1* is downregulated; *Plxnb3* shows potential compensatory upregulation.

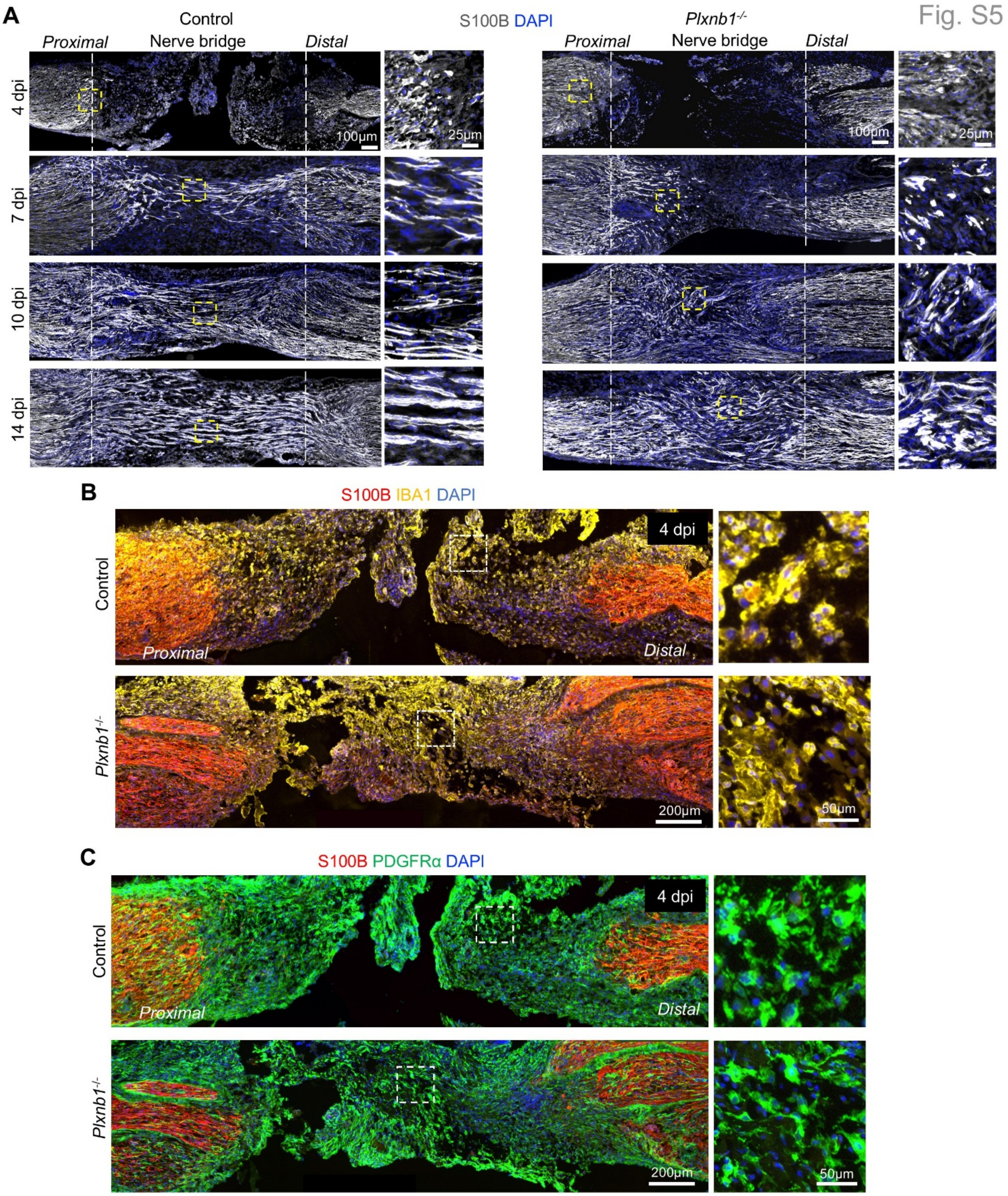

Fig. S5. ***Plxnb1*$^{-/-}$ Schwann cells show spatial disarray and delayed migration into nerve bridge.**

(**A**) Injured sciatic nerves (4-14 dpi) show delayed SC migration and disorganization in *Plxnb1*$^{-/-}$ bridges.

(**B, C**) At 4 dpi, macrophages (IBA1^{+}) and fibroblasts (PDGFRα^{+}) infiltrate the gap between stumps before SCs (S100B^{+}), which largely remain in stumps. Magnified views show similar morphology and random orientation of macrophages and fibroblasts in axon/SC-free gaps in *Plxnb1* mutants and controls.

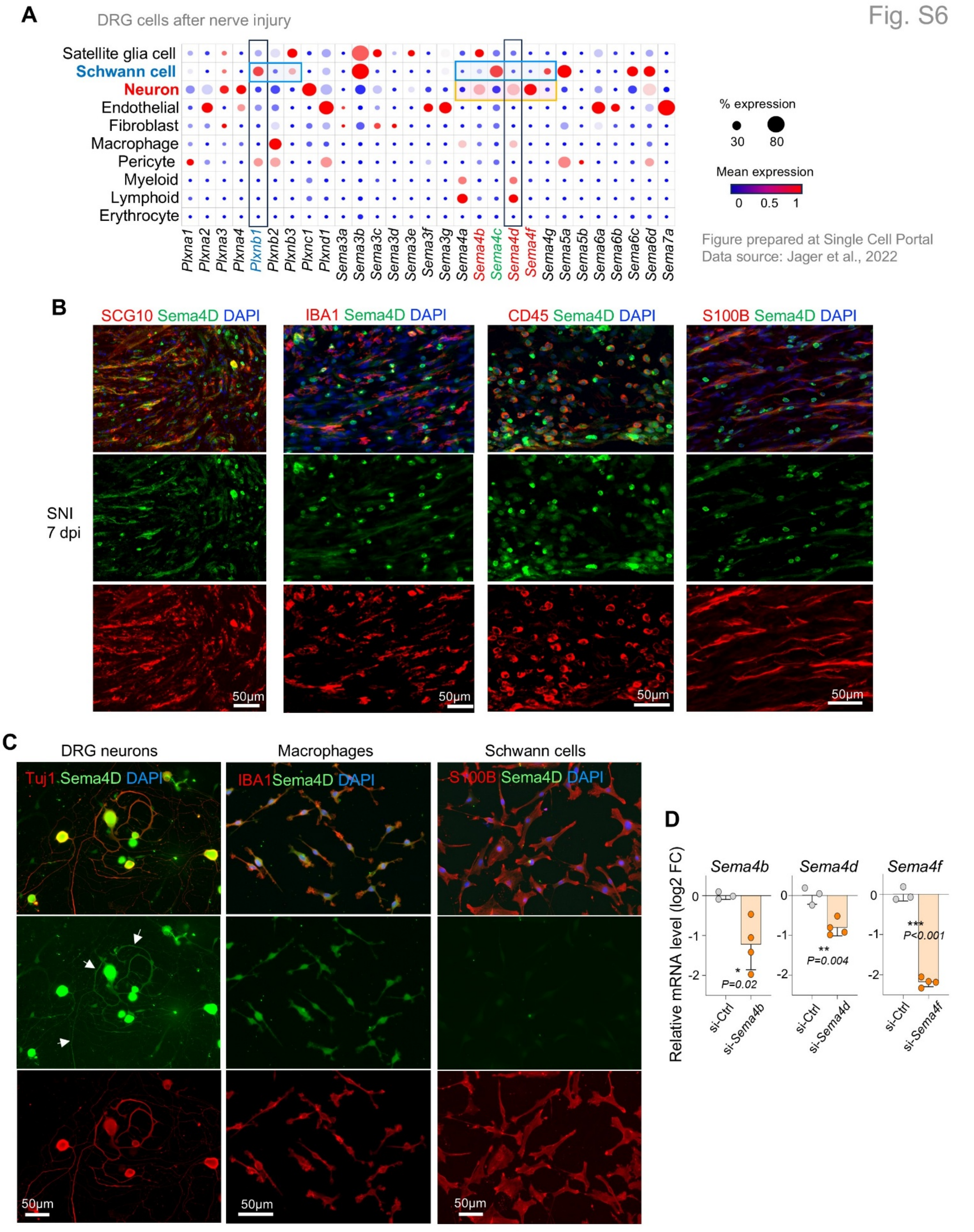

Fig. S6. Expression of Semaphorin-4 genes in injured peripheral nerve.

(**A**) Expression matrix for all Plexin and all Semaphorin genes from DRG single-cell RNA-seq data that was

obtained after nerve injury (Jager et al (*44*)).

(**B**) IF of sciatic nerve at 7 dpi for Sema4D and markers of DRG axons (SCG10), macrophages (IBA1), Schwann cells (S100), or immune cells (CD45).

(**C**) IF of cultured neurons, macrophages, and SCs stained for Sema4D and TUJ1 (neurites), IBA1 (macrophages), or S100B (SCs). Sema4D is detected on cell bodies and on axons (arrows).

(**D**) qRT-PCR of DRG neurons transfected with siRNA against *Sema4* genes confirms efficient knockdown. Mean ± SEM; n = 3 for si-Control, n = 4 for siRNA groups; two-tailed unpaired t-tests.

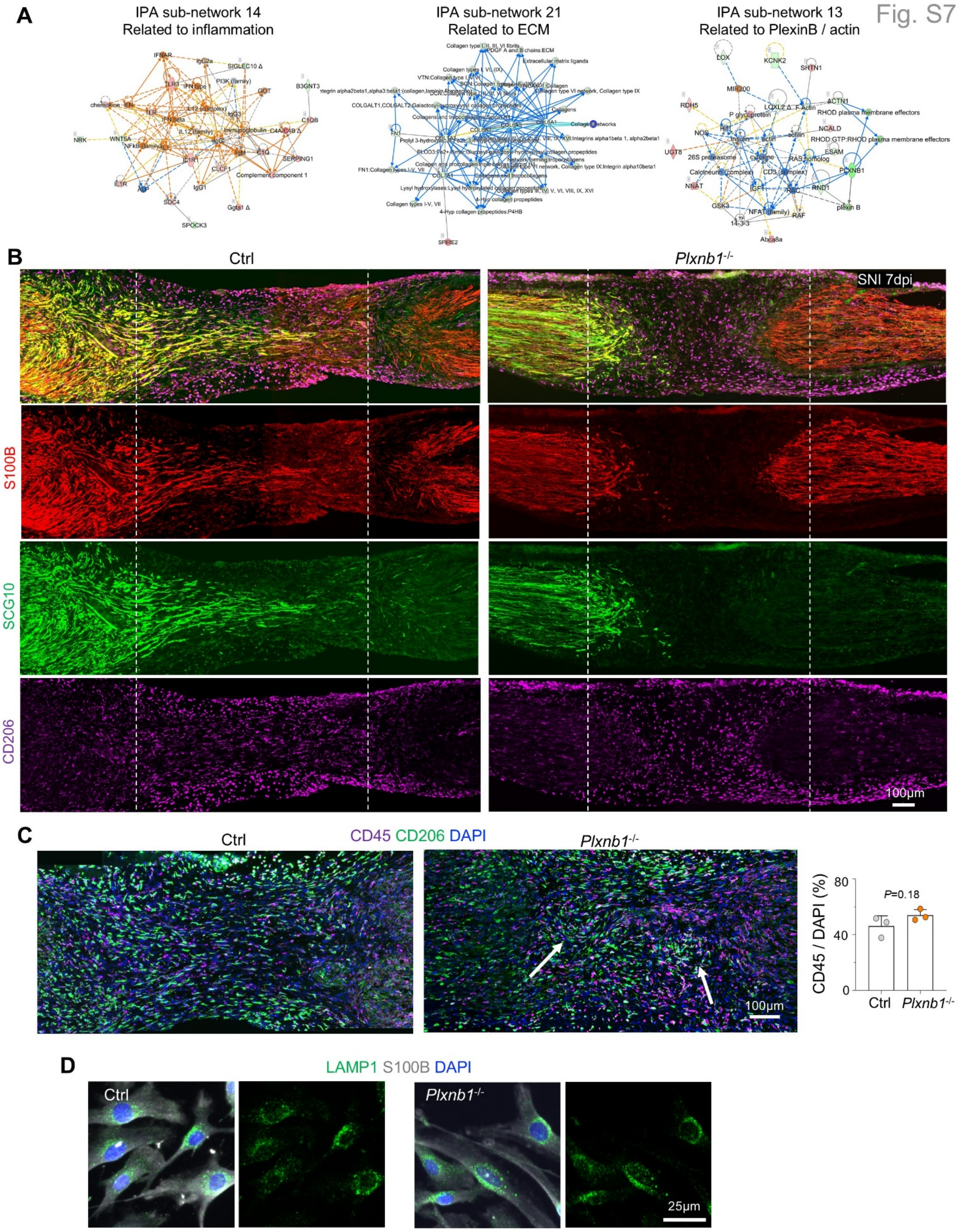

Fig. S7. Plexin-B1 deletion causes spatial disarray and delayed migration of SCs into nerve bridge.

(**A**) IPA subnetworks show predominant activation of inflammation-related genes and inactivation of ECM- and

actin-related genes in *Plxnb1*$^{-/-}$ SCs.

(**B**, **C**) IF and quantification of SCs (S100B^{+}), regenerating axons (SCG10^{+}), and pro-repair macrophages (CD206^{+}) at 7 dpi. In *Plxnb1*$^{-/-}$ nerves, CD206^{+} macrophages cluster proximally and orient vertically to the nerve axis (arrows). Quantifications of CD206^{+} among CD45^{+} cells show no significant differences. Mean ± SEM; n=3; two-tailed unpaired t-tests. The S100B and SCG10 stainings of (B) are also shown in Fig. 6D.

(**D**) IF of control and *Plxnb1*$^{-/-}$ SCs shows similar LAMP1 and S100B expression.

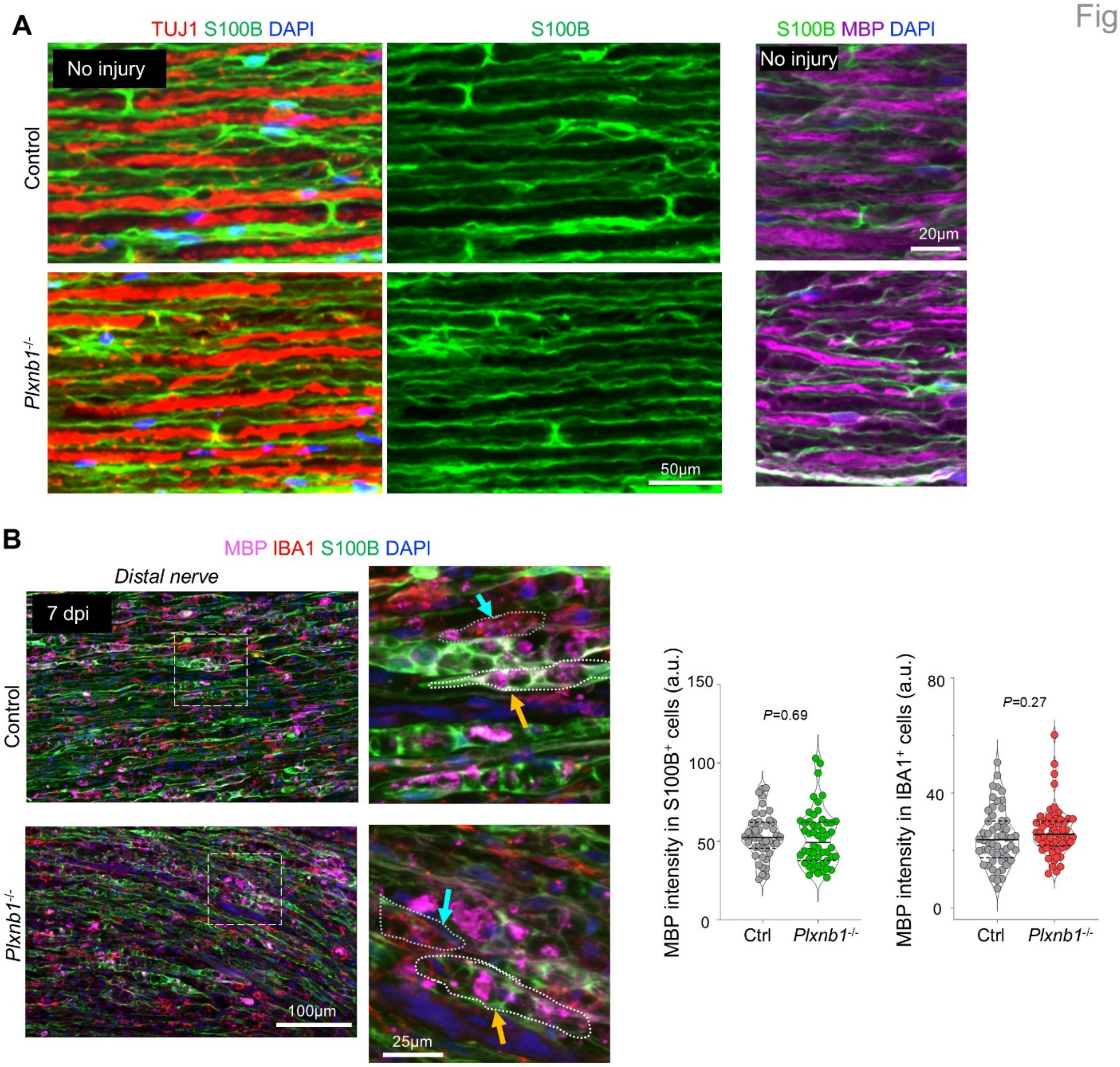

Fig. S8. Plexin-B1 knockout does not affect nerve development or myelination.

(**A**) IF of uninjured sciatic nerves from control and *Plxnb1*$^{-/-}$ adult mice shows similar ensheathment of axons (TUJ1^{+}) by SCs (S100B^{+}) and similar myelination (MBP) levels. DAPI for nuclei.

(**B**) MBP staining in distal nerve stump at 7 dpi shows comparable myelin debris phagocytosed by macrophages (IBA1^{+}, blue arrows) and SCs (S100B^{+}, orange arrows). Violin plots show MBP levels with median and quartiles; S100B^{+}: n=45 (Ctrl), n=52 (*Plxnb1*$^{-/-}$); IBA1^{+}: n=54 (Ctrl), n=60 (*Plxnb1*$^{-/-}$); three mouse pairs; two-tailed unpaired t-tests.

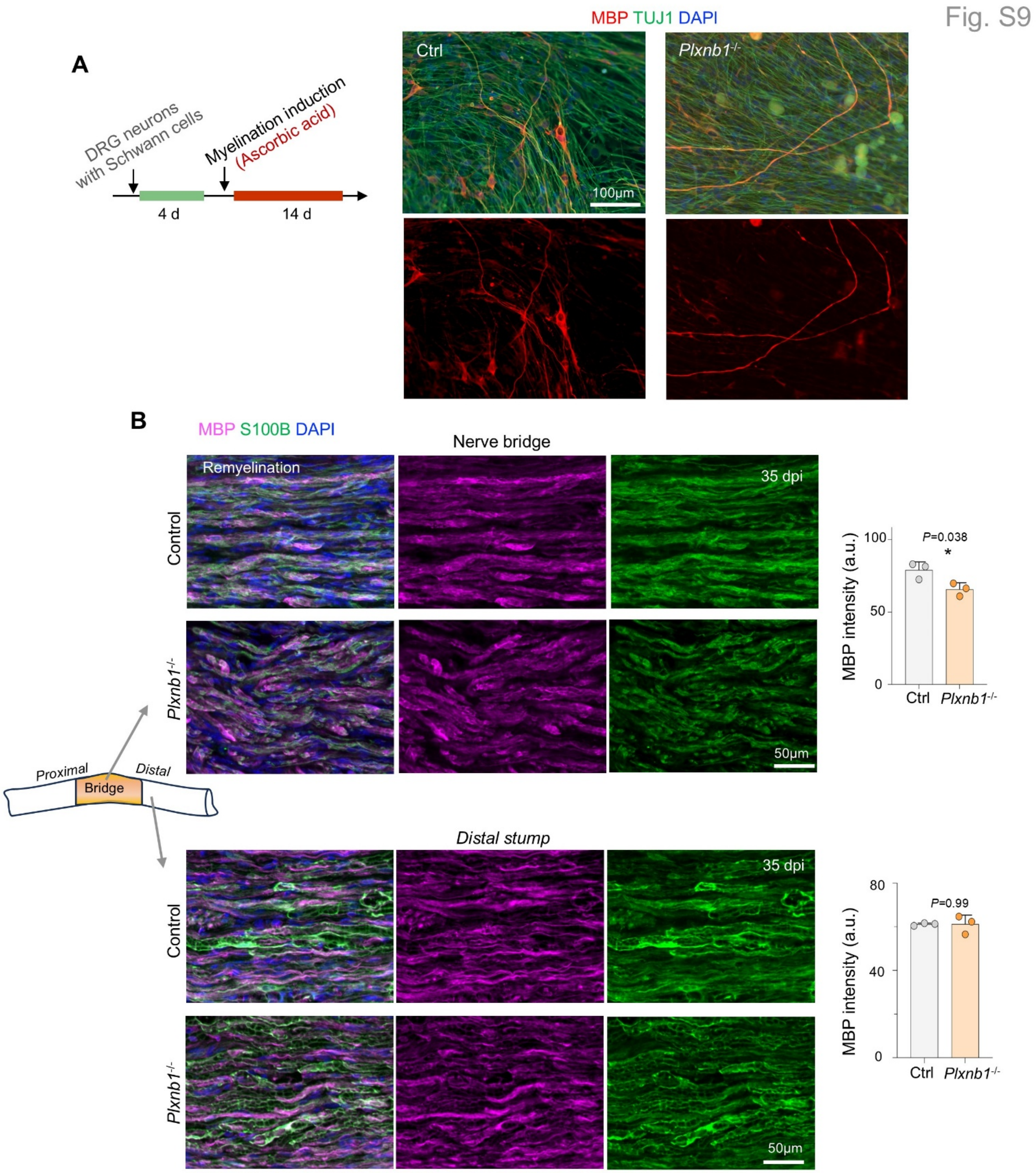

Fig. S9. Myelin breakdown and remyelination are unaffected by *Plxnb1* deletion.

(**A**) Left, timeline of co-culture of adult DRG neurons with SCs from control or $Plxnb1^{-/-}$ mice. Induction of myelination by ascorbic acid. Right, IF shows similar myelin (MBP^{+}) levels in both genotype conditions.

(**B**) IF of sciatic nerves at 35 dpi shows remyelination (MBP^{+}) in both genotypes, with some spatial disarray in the nerves of $Plxnb1^{-/-}$ mice. Mean ± SEM; n=3; two-tailed unpaired t-tests.

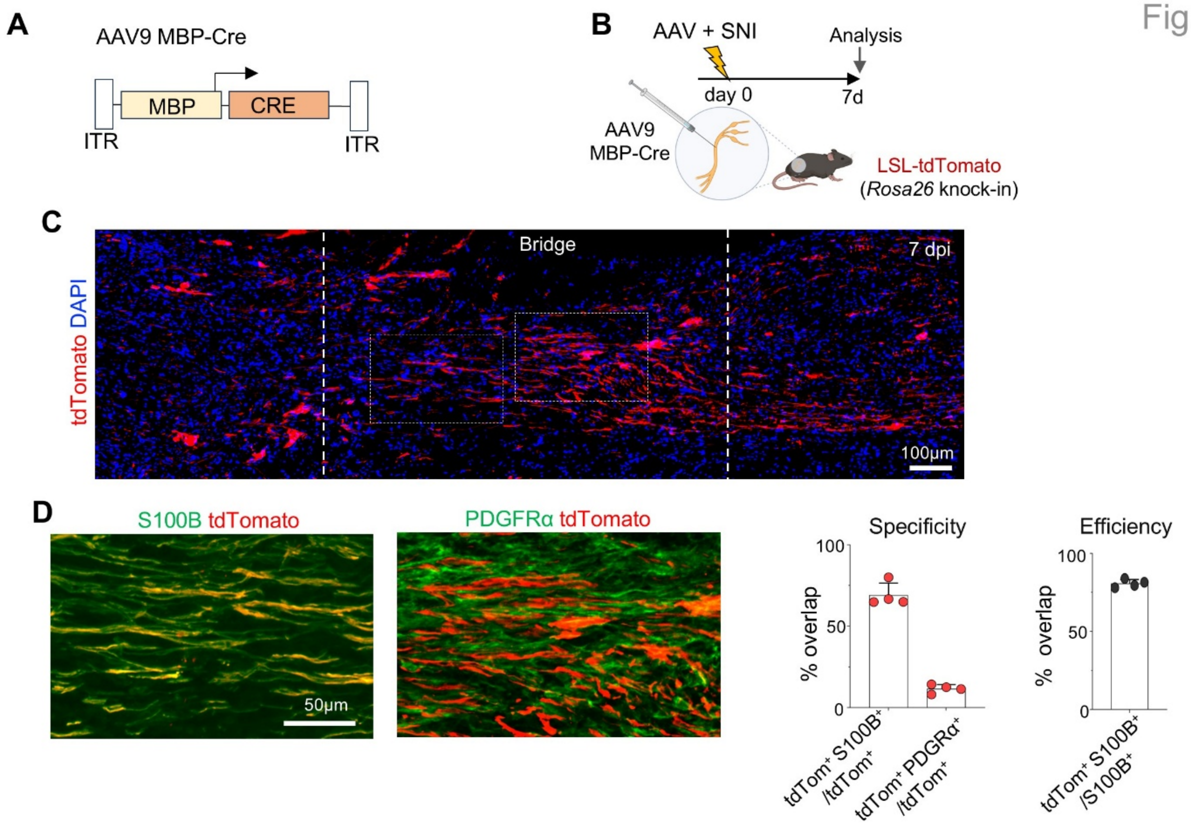

Fig. S10. AAV9 with MBP promoter to target Schwann cells.

(**A**) Diagram of AAV9 vector with myelin basic protein (MBP) promoter to drive expression of Cre recombinase in SCs.

(**B**) Intrasciatic injection of AAV vector into *Rosa26* LSL-tdTomato mice immediately before transection injury. Figure was created in BioRender. Li, J. (2025) https://BioRender.com/m19u012.

(**C**) Fluorescence images from 7 dpi nerves shows tdTomato^{+} cells, enriched in the bridge versus stumps.

(**D**) IF shows high overlap of tdTomato signal with SCs (S100B^{+}) and to a lower degree with fibroblasts (PDGFRα^{+}). Quantification indicates transduction specificity and efficiency for SCs. Mean ± SEM; n=4 bridge fields, two independent experiments.

Legends for Supplementary Tables and Movies

Table S1. DEGs from PB1 KO vs WT SCs.
Differentially expressed genes (DEGs) from RNA-seq of Schwann cells (*Plxnb1*$^{-/-}$ vs. WT). Cutoff: adjusted $P < 0.05$; $|\log_2 FC| > 0.25$.

Table S2. Ingenuity Canonical Pathways.
Ingenuity Canonical Pathways analysis, based on differential gene expression profile of Schwann cells derived from *Plxnb1*$^{-/-}$ vs. wild-type mice.

Movie S1. Schwann cell (WT) live cell imaging.
Live cell imaging of Schwann cells (wild-type), stained with membrane dye NR12 and nuclear dye SPY-DNA. WT SCs display contact inhibition of locomotion.

Movie S2. Schwann cell (PB1 KO) live cell imaging.
Live cell imaging of Schwann cells (*Plxnb1*$^{-/-}$), stained with membrane dye NR12 and nuclear dye SPY-DNA. PB1 KO cells showed trend of clustering together.
